# Flexible response and rapid recovery strategies of the plateau forage *Poa crymophila* to cold and drought

**DOI:** 10.3389/fpls.2022.970496

**Published:** 2022-11-08

**Authors:** Xin-Yu Li, Yan Wang, Xin-Yi Hou, Yan Chen, Cai-Xia Li, Xin-Rong Ma

**Affiliations:** ^1^ Chinese Academy of Sciences, Innovation Academy for Seed Design, Chengdu Institute of Biology, Chengdu, Sichuan, China; ^2^ University of Chinese Academy of Sciences, Beijing, China; ^3^ College of Life Sciences, Sichuan University, Chengdu, Sichuan, China

**Keywords:** *Poa crymophila*, transcriptome-associated metabolome, cold and drought stresses, photosynthetic system, nitrogen transport, polyamine and phenolamide

## Abstract

Cold and drought stress are the two most severe abiotic stresses in alpine regions. *Poa crymophila* is widely grown in the Qinghai–Tibet Plateau with strong tolerance. Here, by profiling gene expression patterns and metabolomics-associated transcriptomics co-expression network, the acclimation of *Poa crymophila* to the two stresses was characterized. (1) The genes and metabolites with stress tolerance were induced by cold and drought, while those related with growth were inhibited, and most of them were restored faster after stresses disappeared. In particular, the genes for the photosynthesis system had strong resilience. (2) Additionally, cold and drought activated hypoxia and UV-B adaptation genes, indicating long-term life on the plateau could produce special adaptations. (3) Phenolamines, polyamines, and amino acids, especially N′,N″,N′″-*p*-coumaroyl-cinnamoyl-caffeoyl spermidine, putrescine, and arginine, play key roles in harsh environments. Flexible response and quick recovery are strategies for adaptation to drought and cold in *P. crymophila*, accounting for its robust tolerance and resilience. In this study, we presented a comprehensive stress response profile of *P. crymophila* and provided many candidate genes or metabolites for future forage improvement.

## Introduction

The cold and arid alpine conditions are the important and governing factors limiting the productivity of grasslands on the Qinghai–Tibet Plateau. The coldest period (November to February) of the Qinghai–Tibet Plateau has a monthly average temperature as low as −20°C~−10°C, and the lowest temperature can reach −30°C (data from China Meteorological Administration, http://data.cma.cn/). On the other hand, precipitation in most parts of the Qinghai–Tibet Plateau is scarce, and precipitation from November to February is even less than 20 mm. Observations from 1980 to 2015 showed a significant increase in the arid area of the Qinghai–Tibet Plateau (Shao et al., 2018). Global climate change exacerbates the occurrence of extreme weather, severely affects vegetation growth, and limits the productivity of prairies.

*Poa crymophila* is a perennial forage species in alpine meadows and grasslands (Shen et al., 2019). Also, the variety *P. crymophila* Qinghai is widely distributed in wet grassland, alpine grassland, forest edges, hillsides, valleys, and beaches at an altitude of 2,150~4,800 m in the Qinghai–Tibet Plateau, including Qinghai, Tibet, and Sichuan provinces, China. After more than 30 years of selection, cultivation, and domestication, the variety was approved by the National Grass Variety Approval Committee in December 2003 (variety registration number 261). This variety not only survives the winter safely at a low temperature even of −36°C with a wintering rate of over 95%, but its well-developed root system is mostly concentrated in the soil layer of 10–18 cm, with a cylindrical root sheath structure that facilitates water absorption from moist sand. It has been cultivated as forage with high tolerance to cold and drought stresses (Zhou et al., 2010; Hua et al., 2019). Therefore, *P. crymophila* is an excellent material for exploring the molecular mechanisms of cold and drought tolerance in the alpine region.

Transcriptome analysis can reveal the mechanism of plant adaptation to stress at the transcriptional level and explore the function of pivotal genes. Weighted gene co-expression network analysis (WGCNA) is an efficient and powerful method for constructing a co-expression network and digging into core genes. In this way, four pivotal genes of *CAD*, *POD*, *CCoAMT*, and *CML* were found in strawberries (*Fragaria nilgerrensis*) under cold stress, which has the key function of controlling high cold resistance (Liu et al., 2021a). Similarly, by comparing with the transcriptome of cold-tolerant and cold-sensitive varieties of Chinese prickly ash (*Zanthoxylum bungeanum* Maxim), the top 150 hub genes were identified and the mechanism of cold stress was discussed (Tian et al., 2021). Furthermore, module preservation analysis (MPA) was developed from the WGCNA algorithm to eliminate irrelevant expression differences and focus on the most variable and relevant unigenes. MPA was used to analyze the molecular mechanism of *Arabidopsis* heterosis by comparing the transcriptomes of ecotypes Col-0, Per-1, and their F1 hybrids, revealing that differentially expressed genes involved in photosynthesis and cell division pathways were the main factors for heterosis (Liu et al., 2021b).

Plant secondary metabolites also play an important role in stress response. For instance, proline could help plant cells maintain redox balance under normal and stressful conditions (Per et al., 2017). Increases in arginine and polyamines were associated with preventing H_2_O_2_ from oxidative stress on the plasma membrane (Hassan and Mohamed, 2019). Phenamides, also commonly known as hydroxycinnamamides (HCAAs), can act as products of polyamine catabolism or as a storage form of polyamines and phenolics, and have specific functions in plant development and environmental adaptation. Some of them, like di-feruloylputrescine, di-feruloylspermidine, and ferulic acid tyramide, were found in the seeds of rice and maize and participated in plant growth by depleting themselves to maintain polyamine levels (Luo et al., 2009). Phenamines are suitable substrates for peroxidases, and they also favor the scavenging of hydrogen peroxide (Groppa et al., 2008); the stress tolerance mainly depends on the antioxidant activity. It was discovered that a special phenamine, *N*′,*N*′′,*N*′′′-*p*-coumaroyl-cinnamoyl-caffeoyl spermidine, can participate in scavenging phenanthrene to protect *Salix viminalis* from poison (Xia et al., 2021).

We reanalyzed previous RT-qPCR-validated transcriptome data combined with metabolome data to explore the source of *P. crymophila’s* robust tolerance to cold and drought. Although many stress-related metabolites and coding genes were found, the regulatory network and factors are still not fully understood. Metabolome-associated transcriptome analysis could further reveal stress resilience mechanisms. Our previous research found that a series of stress tolerance-related metabolites such as phenylpropanoids and carbohydrates, and genes like *MYB* and *CCR*, were found in *P. crymophila* under drought and low-temperature stress (Wang et al., 2021b). In this study, using the advantage of the sophisticated implements of WGCNA and MPA, we further combined the metabolome and transcriptome to deeply explore regulatory networks and reveal the adaptive mechanism of *P. crymophila* to low temperature and drought in an alpine environment.

## Methods

### Material treatments and phenotype analysis

The plants of *Poa crymophila* Keng cv. Qinghai were prepared as the procedure by Wang et al. (2021b). The seeds were planted in plastic pots and grew in a greenhouse for 2 months. Before treatments, plants were transferred to a growth chamber (22/16°C, photoperiod 14 h, at a relative humidity of 60% and an irradiance of 200 mmol·m^−2^ s^−1^) for 2 weeks. The plants grew at 22/16°C and were well-watered as in the control group (CK). For cold treatment, the plants were transferred to another −5°C growth chamber for 24 h (Cold). They were then returned to normal conditions for 48 h of recovery (ReCold). As to drought treatment, the plants were deprived of water for 10 days as part of the drought stress group (Drought) and then rewatered for recovery for 48 h (ReDrought). Three replicates were collected from each sampling point (CK, Cold, Drought, ReCold, and ReDrought), with each replicate containing at least 50 plants. Samples were frozen in liquid nitrogen and stored at −80°C.

The growth state and photosynthetic rate were collected at CK, Cold, Drought, ReCold, and ReDrought time points. The photosynthetic rate was measured using a portable photosynthetic fluorescence measurement system (GFS-3000) with a 3010-standard measuring head and a 3055 leaf chamber fluorometer (Walz, Germany). The measurements were taken from at least four pot plants.

To demonstrate the robust cold tolerance of *P. crymophila*, the cool-season forage perennial ryegrass (*Lolium perenne* cv. Mathilde) with strong cold adaptation was planted at the same time with the same method. *P. crymophila* and *L. perenne* were stressed for 24 h at −5°C and −12°C, recovered for 48 h, and their growth states and photosynthetic rate were compared.

Leaves are not only the main site of photosynthesis but also an important part of sensing adverse conditions. Leaves can directly reflect the growth state of plants. Therefore, we chose leaves as the research material to explore plant stress responses. RNA was extracted and sequenced using an Illumina HiSeqTM 2000 (Illumina) at BGI (Shenzhen, China). The sequencing results with quantitative real-time (qRT)-PCR verification were deposited to the Sequence Read Archive database (http://www.ncbi.nlm.nih.gov/Traces/sra/, accession number: SRX2725266). A widely targeted metabolome was completed by Wuhan Metware Biotechnology Co. Ltd. (Wuhan, China). The results were putatively annotated by the self-built database Metware Database (MWDB; Wuhan Metware Biotechnology Co. Ltd., Wuhan, China). These transcriptome and metabolome data were obtained from preliminary work (Wang et al., 2021b).

### Data filtering, assessment, and differential expression analysis

Based on the terms of the fragments per kilobase of exon model per million (FPKM), the expression levels of unigenes were calculated by RSEM software (RNA-Seq by Expectation Maximization) (Li & Dewey, 2011). The correlation between samples was represented by the square of the Pearson coefficient, and the results were used to reflect the reliability of the experiment and the rationality of sample selection. Statistical parameters such as standard deviation, mean, and coefficient of variation (CV) were used to characterize the expression of unigenes. To filter genes with low expression and poor consistency, genes with an average FPKM < 10 and coefficient of variation (CV) value of biological replicates of > 1 at all samples were discarded. For evaluating the result of data filtering, principal component analysis (PCA) was performed using the PCA OmicShare online tools (https://www.omicshare.com/tools/Home/Soft/pca). The data were normalized by *Z*-core before performing PCA to eliminate the influence of genes with abnormally high and low expression levels.

Based on the unigenes count number, the parameters of expression differences were obtained by R package DESeq2 (Love et al., 2014). The threshold of DEG was set to | log_2_FC|≥1 and false discovery rate (FDR) to <0.05.

### Gene expression pattern and functional enrichment analysis

The expression data were divided into two groups based on the stress types: the cold-related group, which included three sampling points of CK, Cold, and ReCold, and the drought-related group, which included three sampling points of CK, Drought, and ReDrought. The two groups shared the same CK. For profiling the unigene expression pattern upon stress and recovery, the R package TCseq was applied (Jun and Gu, 2021). The expressive pattern of the cold and drought treatment groups was respectively profiled by adopting the “cm” (cmeans) algorithm, setting the *k*-value to “8”, and standardizing it as “True”.

Based on the annotation of the Gene Ontology (GO) and Kyoto Encyclopedia of Genes (KEGG) databases, the selected unigene list was subjected to the online functional enrichment tools (https://www.omicshare.com/tools/Home/Soft/getsoft). Because there is no reference genome for *Poa crymophila*, all unigenes with biological repeat stable expression and high quality were used as the background gene files for the KEGG and GO enrichment analyses.

### Consensus module analysis

For comparing similar features between responses to cold and drought stresses, module preservation and consensus module analysis were performed. Genes with similar functions and shared stress responses can be mined using highly correlated gene modules.

Module preservation analysis was performed between the individually constructed gene co-expression networks of cold- and drought-related datasets with a parameter of 200 nPermutations. The permutation test defines the preservation degree of modules in the two networks by providing Z summary values that summarize the preservation statistics based on density and connectivity. Zsummary > 10 represented strong preservation, 10 < Zsummary < 2 represented moderate to weak preservation, and Zsummary < 2 represented no preservation. By using blockwiseConsensusModules in the R package WGCNA (Zhang B. & Horvath, 2005), the conserved gene co-expression networks affected together by cold and drought stresses were detected. Based on the conserved preservation results, the consensus co-expression network between cold and drought datasets was constructed, and the dynamic tree-cutting method was implemented with the following parameters: maxBlockSize of 15,000, power value of 10, deepSplit level 2, minModuleSize of 30, and mergeCutHeight of 0.25. The co-expression network of each group of core-conserved genes is detected by correlation coefficient (*k*_ME_) to evaluate the value of the module member of each gene. The important metabolic pathways that core genes are involved in were visualized through GO functional annotation.

### Transcriptome-associated widely targeted metabolome analysis

WGCNA, the R package, was implemented for constructing the gene co-expression network. The unigenes were from RNA-Seq data of 15 samples (five sampling points × three replicates) and divided into two groups: the cold-related group (CK, Cold, ReCold) and the drought-related group (CK, Drought, ReDrought) as individual datasets. By setting the maxBlockSize value of 15,000 (both), and the soft threshold power β-value of 18 (cold) and 10 (drought) for balancing intramodule connectivity (**Supplementary Figure S1**) and intermodule independence in different co-expression network, the correlation matrix by cold- and drought-related datasets was transformed into an adjacency matrix, respectively. By using the dissimilarity measure approach, the corresponding topological overlap matrix (TOM) was transited from the two adjacency matrixes. Hierarchical clustering trees were constructed based on TOM similarity for module detection, and each module calculated the relationship with the differential metabolites. Depending on the relationship and *p*-value, the closely related metabolites of each module can be obtained.

According to the annotation of GO, Swissprot, and other databases, the scope of transcriptome analysis was further narrowed, and stress tolerance-related modules and key core genes were identified. The eigenvalues, *k*_ME_, that measure the correlation between each gene and eigengenes of modules, were used to evaluate the core genes of modules. The unigenes with *k*_ME_ *>*0.9 and *p* < 0.001 can be used as core genes representing the expression tendency of modules. Based on all edges of nodes calculated from WGCNA, the co-expression networks were constructed using Cytoscape v3.5.1 (Shannon et al., 2003).

## Results

### The phenotype under low-temperature and drought stress

**Figure 1:**
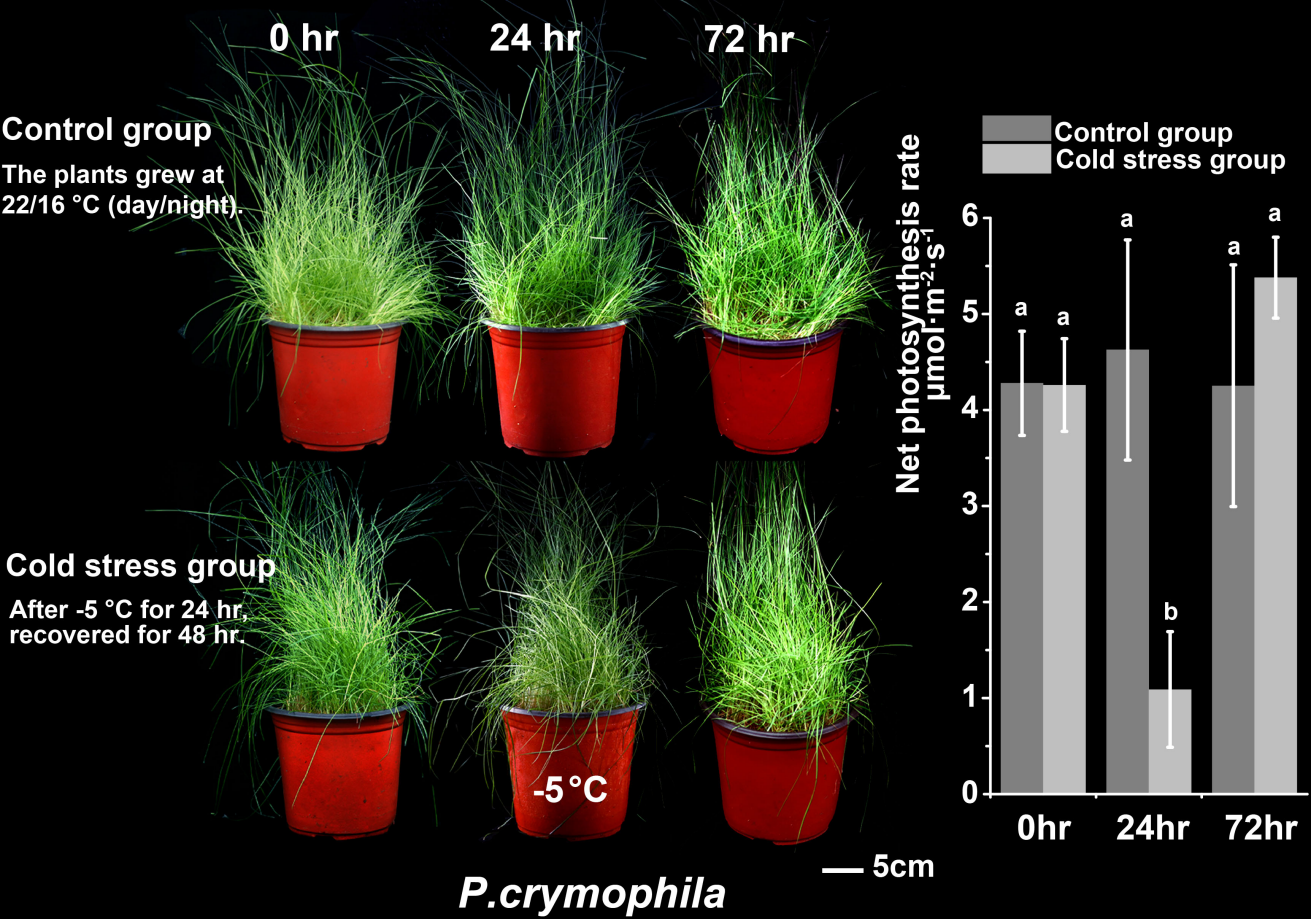
The cold stress (−5°C) and recovery of *P. crymophila*.

**Figure 2:**
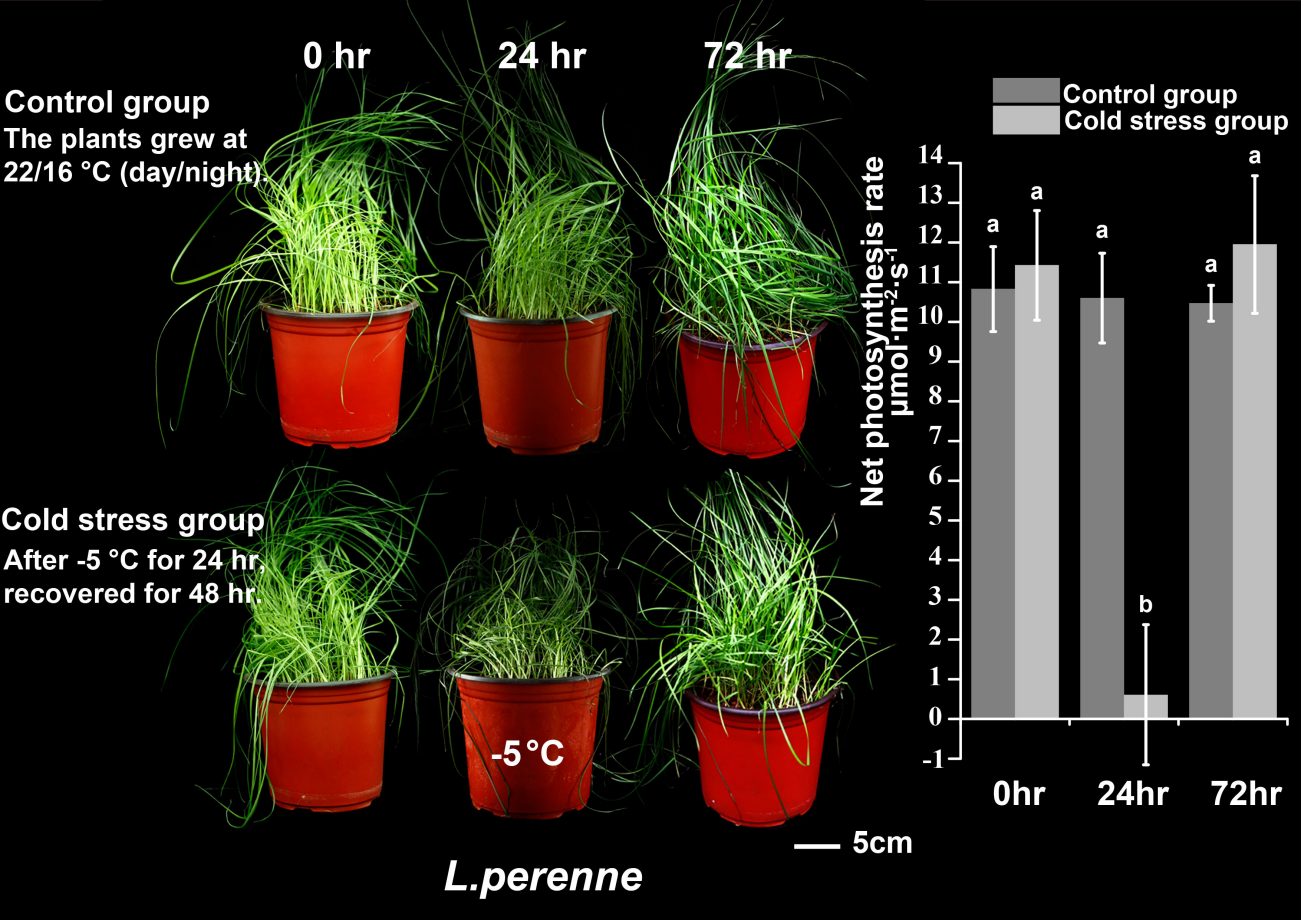
The cold stress (−5°C) and recovery of *L. perenne*.

**Figure 3:**
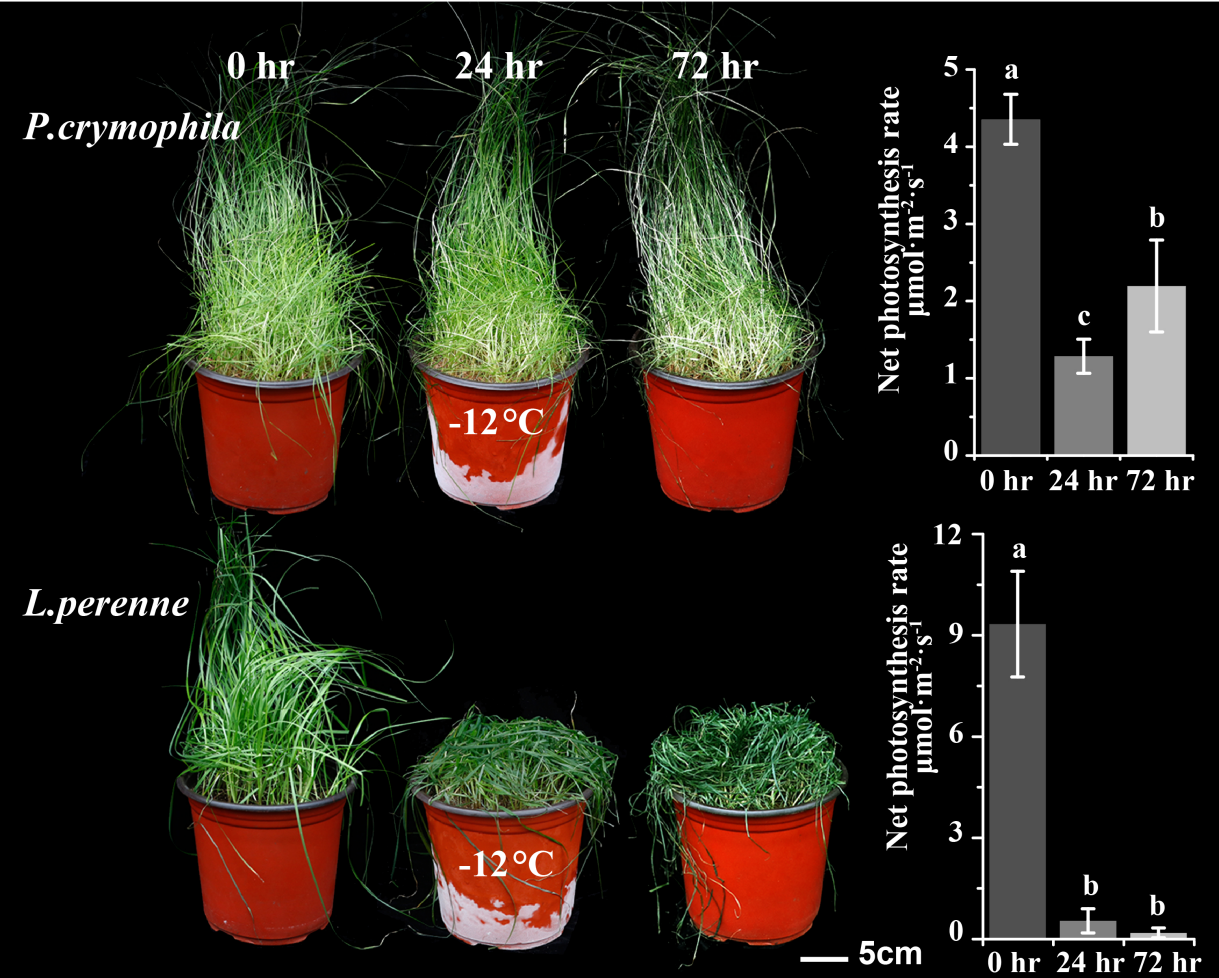
The cold stress (−12°C) and recovery of *P. crymophila* and *L. perenne*.

We subjected *P. crymophila* to low-temperature stress at −5°C and −12°C, as well as drought stress for 10 days after water was cut off, before recovering for 48 h. Simultaneously, *L. perenne* was treated at the same low temperature to compare the frost resistance of *P. crymophila*. The results showed that the growth of *P. crymophila* and *L. perenne* was inhibited under a low temperature of −5°C, and their photosynthetic efficiency also decreased significantly. However, after 48 h of recovery, the plant can completely recover and the photosynthetic index reaches the control level (**Figures 1**, **2**). Furthermore, under low-temperature stress of −12°C, *P. crymophila* survived after freezing, and its growth gradually recovered after 48 h, with the net photosynthetic rate returning. Regardless, *L. perenne* was damaged by frost at −12°C to death (**Figure 3**).

**Figure 4:**
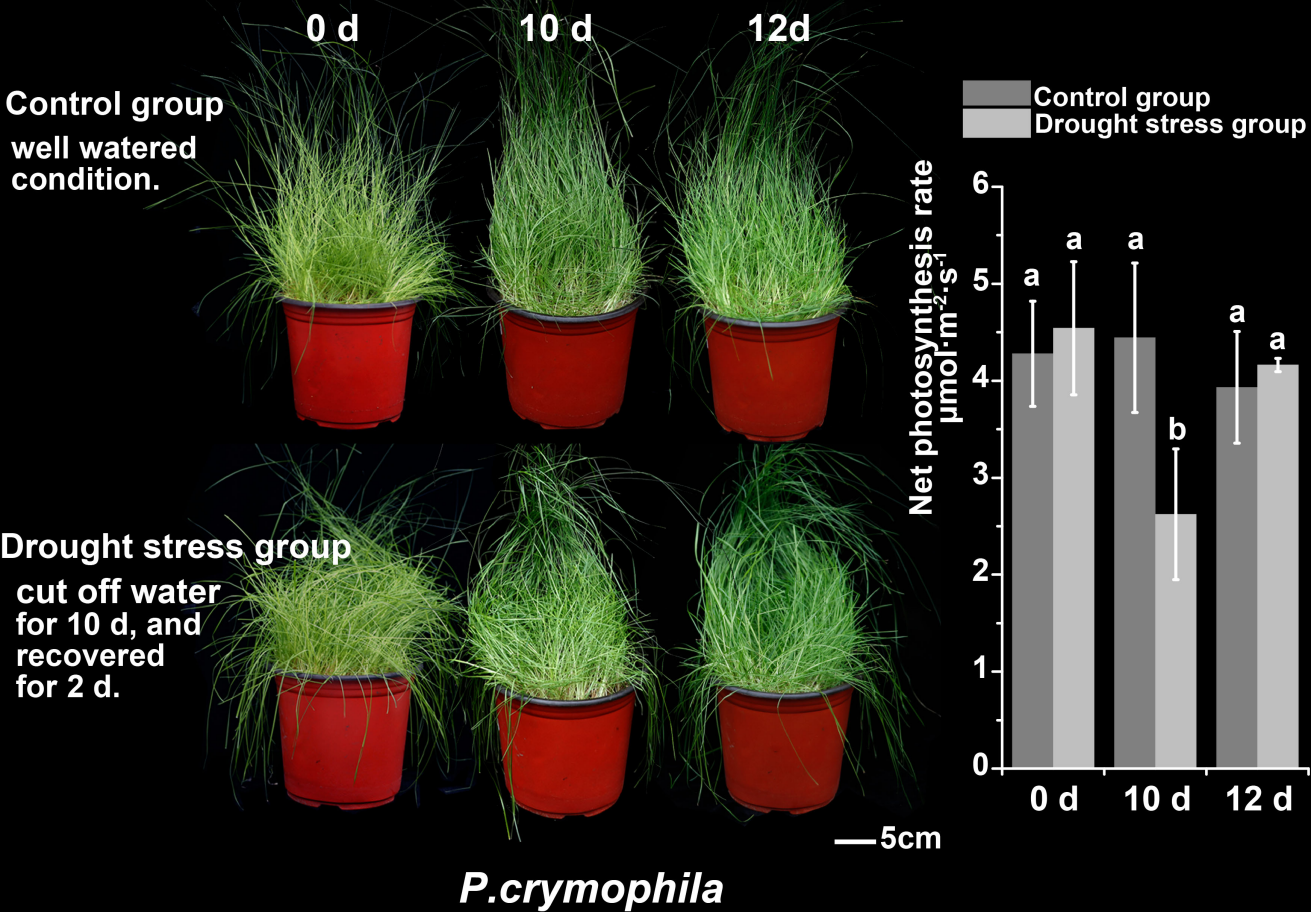
The drought stress and recovery of *P. crymophila*.

Ten-day drought stress has little impact on *P. crymophila*, and its growth status and photosynthetic rate have quickly returned to normal after 48 h of rehydration (**Figure 4**).

### Low-temperature- and drought-responsive transcriptome

A Pearson correlation analysis indicated that the three biological replicates of each treatment showed highly consistent transcriptome profiles (*R* ^2^ = 0.8192–0.9906, **Supplementary Figure S2**). Based on previous research, further focus should be on core differentially expressed genes, with low-expressed and poorly consistent genes being excluded. Genes with an average FPKM of <10 and a CV of biological replicates of > 1 were discarded at all sampling points, leaving 14,380 genes. All of them exhibited high consistency between biological replicates and apparent partitions between different treatments (**Supplementary Figure S3**; **Supplementary Table S1-1**).

A total of 4,577 nonredundant DEGs from the cold-related group were identified (**Supplementary Figure S4A**; **Supplementary Table S1-2**), including 2,599 DEGs (1,679 upregulated, 920 downregulated) in Cold *vs*. CK, 1,983 DEGs (1,202 upregulated, 781 downregulated) in ReCold *vs*. CK, and 2,662 DEGs (1,153 upregulated, 1,509 downregulated) in ReCold *vs*. Cold. In total, 5,129 nonredundant DEGs were obtained from the drought-related group (**Supplementary Figure S4B**), including 2,032 DEGs (933 upregulated, 1,099 downregulated) in Drought *vs*. CK, 2,623 (1,434 upregulated, 1,189 downregulated) in ReDrought *vs*. CK, and 3,087 DEGs (1,838 upregulated, 1,249 downregulated) in Redrought *vs*. Drought.

### Gene expression pattern and function analysis

**Figure 5:**
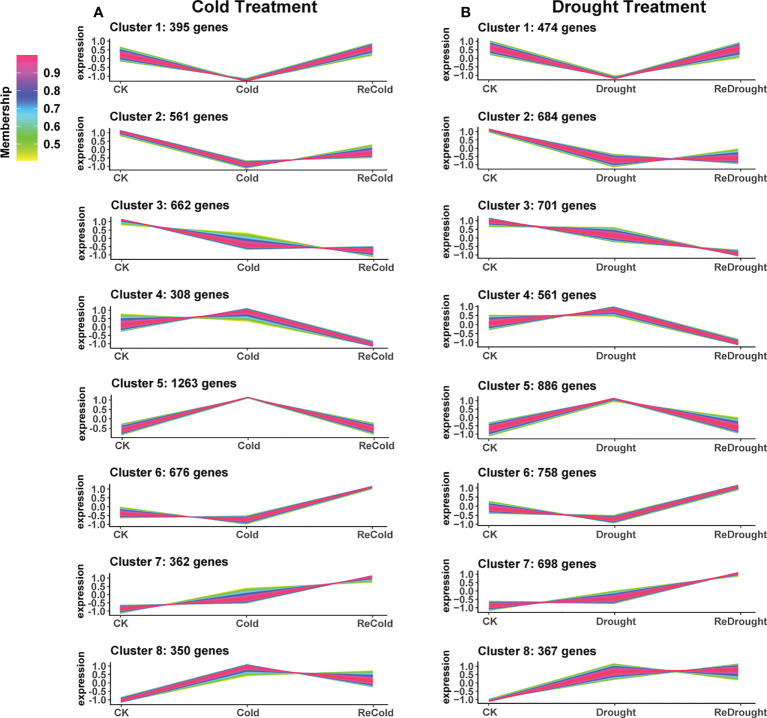
The expression patterns of differentially expressed genes. **(A)** Cold stress. **(B)** Drought stress. The vertical axis represents the relative level of the gene, and the line color represents the model fit.

In order to explore the mechanism of cold and drought stress adaptation, gene expression pattern analysis was performed. As a result, 4,577 cold-responsive DEGs and 5,129 drought-responsive DEGs were all clustered into eight expression patterns, clusters 1~8 (**Figure 5**; **Supplementary Table S2**). The expression of DEGs in cluster 1 was decreased by stress and then recovered to the control level after stress removal. Cluster 2 was downregulated and then recovered only slightly. Cluster 3 was reduced and difficult to recover. Cluster 4 was unchanged under stress but downregulated during recovery. Cluster 5 was stress-induced and quickly returned to normal levels. Cluster 6 was induced only during recovery. Cluster 7 was induced by stress and consistently upregulated during recovery. Cluster 8 was stress-induced and recovered slightly but not to control levels. Based on the functional analysis of GO annotations, the different clusters were involved in different processes (**Table 1**). Most abiotic stress response genes were found in clusters 5, 7, and 8, while clusters 1, 2, and 3 were mainly related to plant growth. Clusters 4 and 6 had a specific response that was only down- or upregulated after the stress disappeared, with the former being associated with negative effects on plants and the latter with the photosynthetic system and other positive effects.

**Table 1:** The gene functions of different patterns in response to cold or drought.

Cluster	Expression pattern	Cold stress		Drought stress	
		Function	Representative genes	Function	Representative genes
1	Decreased and then recovered to control level	Water, glycerol, carbohydrate, nitrogen transport; anther morphogenesis; plant epidermis development	NRT1.1, RCAA, PIP1-2, CHLP, LHCA4	Nitrogen transport; photosynthesis; photosynthetic electron transport chain; oligopeptide transport	NRT1.1, MT2C, PSBT, PSAF, CAO
2	Decreased and then recovered, but only slightly	Auxin polar transport; flower morphogenesis; pollen–pistil interaction; regulation of protein ubiquitination	ABCB21, D3, COL6, RPT2, PAO	Malate transmembrane transport; protein deacetylation; regulation of root morphogenesis; callose deposition in cell wall	RPT2, PSY, CML10, ASG4
3	Decreased and difficult to recover	Meristems transition from vegetative to reproductive stages; photoperiodism; circadian rhythm; protein modification; seed dormancy; seedling development process	UBC28, FH7, FH20, DI19-1, HSC-2, CSN1	Regulation of biological process (nitrogen compound, nucleobase-containing compound, metabolic process, nucleic acid-templated transcription)	UBC28, AGL19, ARR2, IAA1, IAA6, IAA10
4	Decreased only during recovery	Histone H2B ubiquitination; negative regulation of developmental process (flower, post-embryonic and other developments)	UBC15, UBC2, MADS22, SPA4, SIZ1	Superoxide anion generation; purine nucleobase catabolic process; multiple biological processes	PP2C30, CIPK14, XDH, MCSU3, MADS22
5	Increased and then rapidly returned to normal levels	Cold acclimation; polyamine; carbohydrate biosynthetic process; regulation of response to osmotic stress	COR410, LEA14-A, NAC67, LTI6B, NRT1.3, DREB1H	Response to desiccation; L-proline biosynthetic process; carbohydrate homeostasis; stomatal movement	LEA14-A, NAC67, P5CS, CAT2, NRT1.3, COR413P
6	Increased only during recovery	Lipid, carbohydrate derivative, nitrogen compound, and vitamin metabolic process; monosaccharide transport; photosystem II assembly; plastid organization	LHBC, CAB1, CAB8, PETE, WHAB1.6, LHCA4	Photosynthesis (light harvesting); chlorophyll and carotenoid biosynthetic process; terpenoid, phospholipid, polysaccharide organonitrogen biosynthetic process	LHBC, CAB1, CAB8, PETE, LHC, RCABP89
7	Consistently increased	Photosynthesis (light capture and dark reactions); ammonia assimilation cycle; isopentenyl diphosphate; organonitrogen compound metabolism	RCABP89, LHBC, CAB1, CAB1B, GAD	Photosystem assembly; protein targeting to chloroplast; other chloroplast organization processes	PSBS, GAPC2, CAB1, TGA4, WHAB1.6
8	Increased and then recovered slightly, but not to control levels	Response to UV-B, karrikin, disaccharide, red light; nicotinamide nucleotide and inositol phosphate metabolism	HY5, UGT73C5, PSBS, PAP2	Similar to cluster 7	GAPC, SODCP, CAB7, UVR8

### Consensus co-expression network between cold and drought

**Figure 6:**
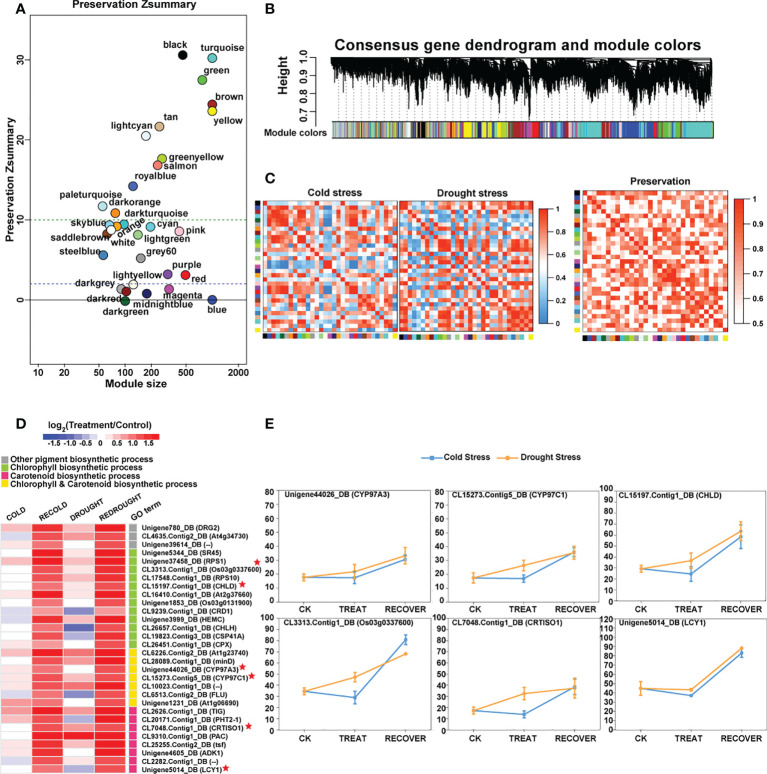
The preservation of consensus modules (CMs) and the functions of CM core genes. **(A)** The preservation results of CMs. The vertical axis represents the *Z*-score, and the horizontal axis denotes the module size. **(B)** Hierarchical cluster dendrogram showing consensus gene co-expression modules. In the dendrogram, each leaf represents one gene, and each module below the dendrogram is labeled with one color. **(C)** Heat maps of eigengene networks showing the relationships among CMs in cold and drought stress. The preservation heat map shows the preservation network, defined as one minus the absolute difference of the eigengene networks in both sets, and the barplot depicts the mean preservation of adjacency for each of the eigengenes to all other eigengenes. **(D)** The expression profile of the important core genes. In the heat map, color intensity represents the coefficients calculated by expression pattern analysis, and different GO terms are represented by different colors. Some genes marked with red stars as the characterization of each GO term show expression trends in line graphs. **(E)** The line graphs of some function genes with a star mark related to photosynthetic pigment biosynthesis. Drought stress is represented by a yellow line, and cold stress is shown by a blue line. The horizontal axis denotes the sampling points, and the vertical coordinates reflect the gene expression level (FPKM value).

To find the similar and strongly correlated responses and genes under the cold and drought stresses, the consensus co-expression network between both of them was constructed using MPA analysis. The results were represented by consensus modules (CMs), indicating the clusters of co-expressed genes. As a result, 14,342 genes were assigned to 31 non-grey CMs (**Figure 6B**; **Supplementary Table S3-1**).

The conservation of co-expression networks with each other was assessed using permutation tests (**Figure 6A**). By projecting the drought-related transcriptome dataset onto the cold-related network, 12 out of 31 modules were strongly preserved (Z_summary_ > 10) and 11 modules exhibited weak to moderate preservation (10 > Z_summary_ > 2). A total of 23 out of 31 modules were at least weakly preserved in drought compared with cold stress (**Supplementary Table S4**). These results suggested that some similar and conserved transcriptional architecture dynamically regulated by cold and drought may exist in *P. crymophila* (**Figure 6C**).

Among those preserved modules, some genes with similar responses to both cold and drought stress were discovered. Abiotic stress response genes *LTI6B* and *LTI65* and transcription factors *NAC67* and *DREB1B* were downregulated and then recovered to the control level. While growth-related *PKS3*, *ARF17*, and *APC7* were suppressed by stress, their expression levels were completely or partially restored after the stress disappeared. In addition, as the members of the NRT1/PTR family are involved in nitrogen transport in plants, the members of *NRT1.1* rapidly recovered after being inhibited by stress, while the *NRT1.3* genes, on the contrary, rapidly decreased after being activated by stresses, and *NRT1.4*s showed significant upregulation during plant recovery. These genes had similar response patterns in response to the two stresses and participated in important biological functions, which may account for the general strategies for cold and drought response.

**Figure 7:**
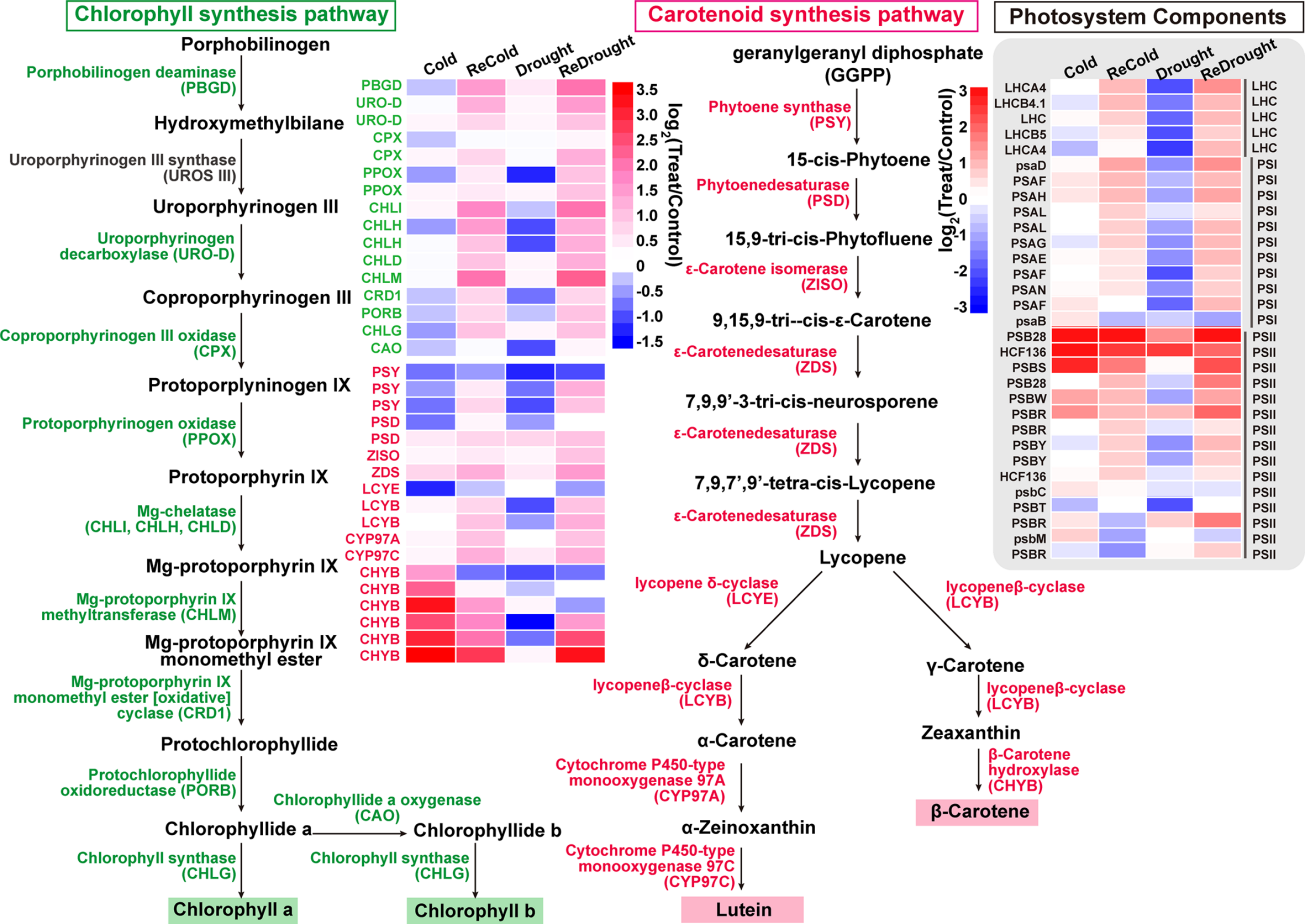
The components of photosynthetic pigment biosynthesis and photosystem. The expression changes of genes are denoted by heat maps, and the color intensity represents the expression level.

Additionally, based on thresholds (*k*_ME_ in cold set or drought set > 0.9 and *p* < 0.001), a total of 3,859 core genes were submitted to GO enrichment analysis (**Supplementary Table S3-2**). Moreover, the results showed that these genes were significantly enriched in photosynthetic pigments (chloroplasts and carotenoids) and were found in the chloroplast stroma, thylakoid, and envelope (**Table 2**). Of them, CYP97A3, CYP97C1, CHLD, etc., were only significantly upregulated during the recovery process (**Figures 6D, E** **)**. Furthermore, 34 enzyme genes involved in chlorophyll and carotenoid synthesis were found (**Figure 7**). Most of them had similar responses that were slightly inhibited by stress and recovered quickly after the stress disappeared. Only CHYB responded differently under the two stressors. Cold stress upregulated their expression but drought inhibited them. Moreover, we also found 31 genes encoding photosystem proteins, such as *LHCs*, *PSIs*, and *PSIIs*, play important roles in photosynthesis. Both *LHC*s and *PSI*s were inhibited by cold and drought stress, especially under drought stress. They were also able to return to normal levels after the stress disappeared. Although *PSII*s responded differently to stress, most were upregulated during the recovery process.

**Table 2:** The GO functions of the core genes in consensus modules between cold and drought.

ID	Description	Nonredundant DEGs	FDR	Cold stress		Drought stress	
				DEG number	Main expression patterns	DEG number	Main expression patterns
GO:0006779	Porphyrin-containing compound biosynthetic process	31	5.07E−05	26	Cluster 6 (20)	29	Cluster 7 (17)
GO:0033014	Tetrapyrrole biosynthetic process	31	8.81E−05	26	Cluster 6 (20)	29	Cluster 7 (17)
GO:0019288	Isopentenyl diphosphate biosynthetic process, methylerythritol 4-phosphate pathway	41	5.01E−06	37	Cluster 6 (31)	39	Cluster 7 (22)
GO:0009240	Isopentenyl diphosphate biosynthetic process	41	5.01E−06	37	Cluster 6 (31)	39	Cluster 7 (22)
GO:0046490	Isopentenyl diphosphate metabolic process	41	5.01E−06	37	Cluster 6 (31)	39	Cluster 7 (22)
GO:0046148	Pigment biosynthetic process	48	3.08E−06	39	Cluster 6 (28)	43	Cluster 7 (21)
GO:0009658	Chloroplast organization	40	9.44E−05	34	Cluster 6 (26)	39	Cluster 7 (20)
GO:0019682	Glyceraldehyde-3-phosphate metabolic process	57	5.01E−06	48	Cluster 6 (38)	54	Cluster 7 (28)
GO:0042440	Pigment metabolic process	54	8.10E−06	43	Cluster 6 (28)	49	Cluster 7 (23)
GO:0008299	Isoprenoid biosynthetic process	57	5.01E−06	46	Cluster 6 (37)	53	Cluster 7 (24)
GO:0006720	Isoprenoid metabolic process	59	5.97E−06	40	Cluster 6 (30)	45	Cluster 7 (21)
GO:0009532	Plastid stroma	118	1.99E−09	92	Cluster 6 (62)	114	Cluster 7 (57)
GO:0009570	Chloroplast stroma	115	2.35E−09	90	Cluster 6 (60)	111	Cluster 7 (55)
GO:0009579	Thylakoid	85	7.76E−06	65	Cluster 6 (47)	83	Cluster 7 (42)
GO:0009941	Chloroplast envelope	93	6.03E−05	75	Cluster 6 (46)	91	Cluster 7 (40)
GO:0009526	Plastid envelope	94	8.02E−05	76	Cluster 6 (46)	92	Cluster 7 (41)
GO:0044435	Plastid part	184	5.73E−09	141	Cluster 6 (92)	178	Cluster 7 (85)
GO:0044434	Chloroplast part	181	9.36E−09	139	Cluster 6 (90)	175	Cluster 7 (83)
GO:0009507	Chloroplast	300	8.39E−07	221	Cluster 6 (134)	282	Cluster 7 (116)
GO:0009536	Plastid	396	8.65E−06	298	Cluster 6 (160)	356	Cluster 7 (132)

### Transcriptome-associated metabolome co-expression network

The association analysis of transcriptome and metabolome was performed by WGCNA. Based on the correlation between transcriptome and metabolome at different sampling points, unigenes can be divided into several modules (**Supplementary Figures S5**, **S6**). Genes in the same module may have similar features or highly interrelated functions. Through analyzing the key modules, their highly related metabolites also can be found. In this way, we can better understand the response of *P. crymophila* to cold and drought stress.

**Figure 8:**
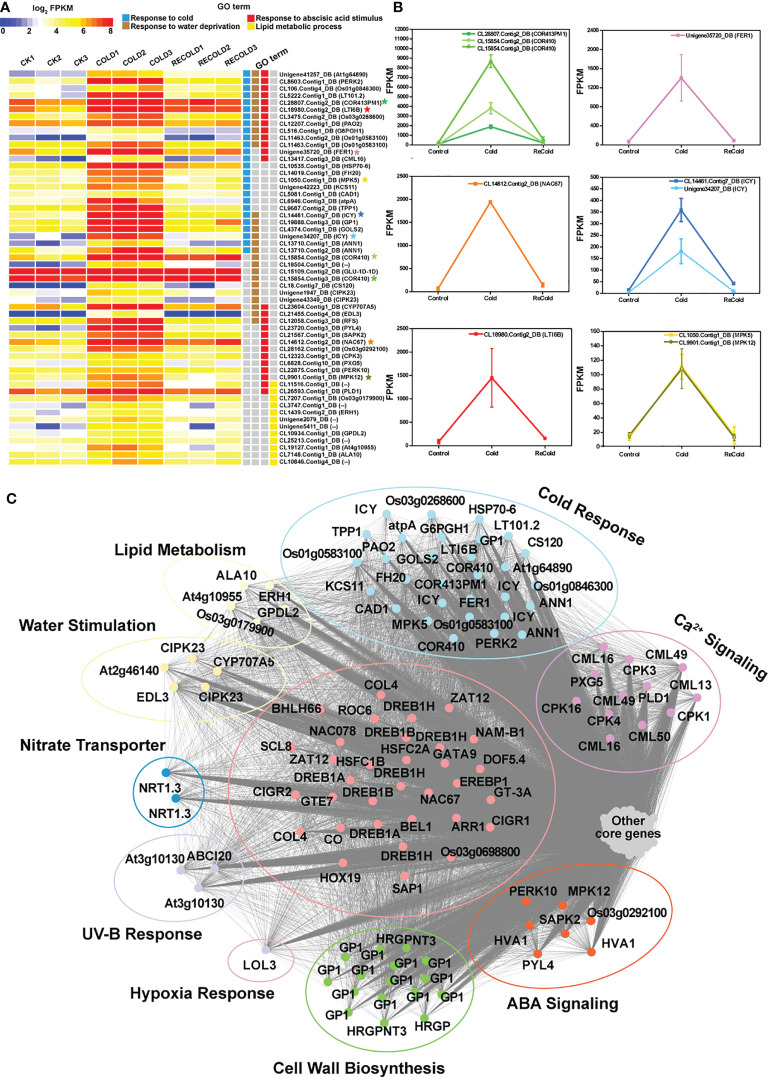
The expression patterns and functions of CMEblue core genes under cold. **(A)** Expression profiles of the representative CMEblue genes (**Supplementary Figure S3**). In the heat map, each gene has three stages and three repetitions. Color intensity represents the expression level, and different GO terms are represented by four columns with different colors. Some genes marked with stars as the characterization of GO term show expression trends in line graphs. **(B)** The line graphs of some function genes with a star mark related to the cold stress response. The horizontal axis denotes the sampling points, and the vertical coordinate reflects the gene expression level (FPKM value). **(C)** Different functions of CMEblue hub genes. The edges represent potential relationships between genes, and the thicker the line, the stronger the correlation.

In cold-related datasets (CK, Cold, and ReCold), 14,376 genes were divided into 25 cold-related modules (CMEs) (**Supplementary Figure S5**; **Supplementary Table S5-1**). Among these CMEs (**Supplementary Figure S5**), only the 1,563 unigenes in CME-blue responded positively to cold stress and had the coldest acclimation-related genes, such as *COR413PM1*, *ICY*, *LTI6B*, *CAD1*, *CML16*, etc. (**Figure 8A**). Based on the threshold of *k*_ME_ ≥0.9, a total of 525 core unigenes were identified from the 1,563 unigenes in CME-blue. These core unigenes respond to cold and are involved in lipid metabolism and response to water deficiency, abscisic acid, UV-B, etc. (**Figure 8C**). In addition, a total of 33 transcription factors like *NAC67* (*CL14612.Contig2_DB*), *DREB1As* (*CL7116.Contig8_DB*, *Unigene43646_DB*), *DREB1B* (*CL26928.Contig10_DB*), and *HSFC1B* (*CL6331.Contig3_DB*) with important regulatory functions were located in the core of CME-blue. Therein, *NAC67* (*CL14612.Contig2_DB*) increased from 67 to 1,793 by cold and then recovered to 146 (**Figure 8B**). Therefore, CME-blue may play a crucial role in cold stress tolerance, and its associated metabolites can be inferred to have similar tolerant functions.

These 25 CMEs had a different relationship with 213 metabolites with significant changes (**Supplementary Figure S7**; **Supplementary Table S6-1**). Based on |Correlation|>0.8, *p* < 0.05, 195 metabolites highly related to CME-blue were identified (**Supplementary Figure S7**; **Supplementary Table S6-1**). In the 90 metabolites with positive correlation, there are 27 lipid compounds (LysoPC 16:1, 9-HOTrE, 14,15-dehydrocrepenynic acid, etc.), 12 flavones (C-hexosyl-chrysin *O*-feruloylhexoside, afzelechin, phloretin, etc.), eight amino acids and derivatives (arginine, asparagine, homocysteine, glutathione oxidized, etc.), six carbohydrates (d-glucose 6-phosphate, glucose-1-phosphate, d-fructose 6-phosphate, etc.), five nucleotide and derivates (hypoxanthine, uridine 5′-monophosphate, hypoxanthine-9-β-d-arabinofuranoside, etc.), five organic acids (azelaic acid, γ-aminobutyric acid, creatine, etc.), and others (nandrolone, azadiradione, dehydrovomifoliol, etc.). In addition, 105 negatively correlated metabolites contained 45 flavonoids, 16 phenylpropanoids, 15 organic acids and derivatives, and others. Among them, syringetin, baicalin, isorhamnetin 3-*O*-neohesperidoside, and isorhamnetin *O*-acetyl-hexoside were the most inhibited.

**Figure 9:**
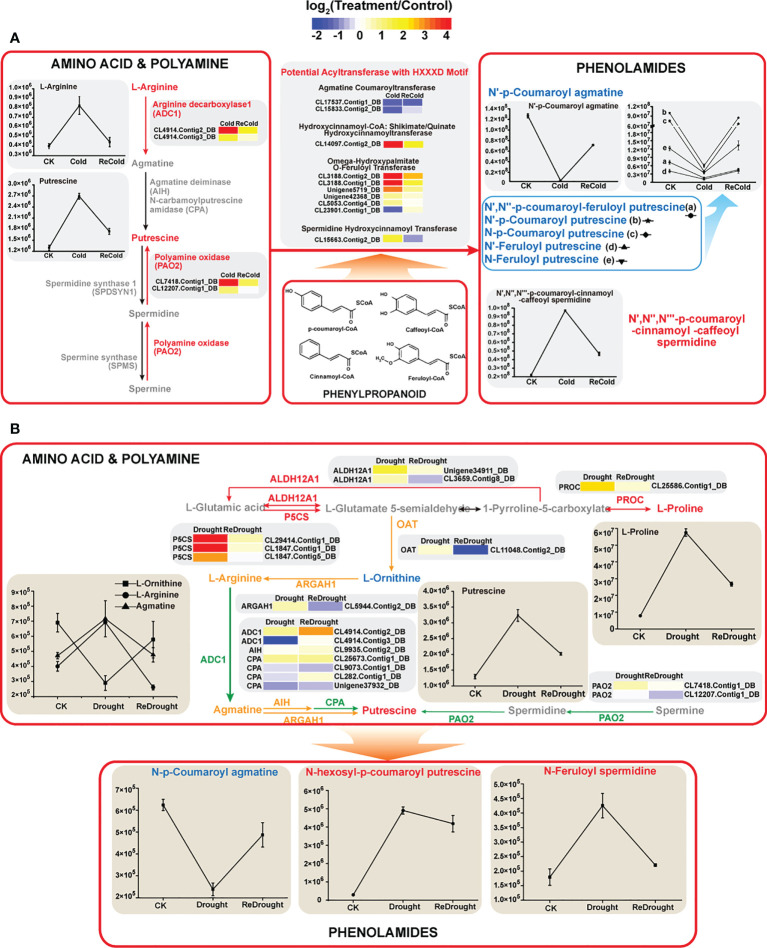
Amino compound metabolism under cold or drought stresses. **(A)** Cold stress. **(B)** Drought stress. The expression changes of genes are denoted by heat maps, and the color intensity represents the expression level. The fluctuant contents of metabolites are represented by line charts. Red arrows and labels: genes or metabolites significantly upregulated; yellow arrows and labels: genes or metabolites moderately upregulated; green arrow and label: gene members with up- or downregulated changes. ALDH12A1, delta-1-pyrroline-5-carboxylate dehydrogenase 12A1; P5CS, delta-1-pyrroline-5-carboxylate synthase; PROC, pyrroline-5-carboxylate reductase; OAT, ornithine aminotransferase; ARGAH1, arginase; ADC, arginine decarboxylase; AIH, agmatine deiminase; CPA, *N*-carbamoylputrescine amidase; PAO_2_, polyamine oxidase 2.

In addition to our previous findings of sugars, flavonoids, phospholipids, etc. (Wang et al., 2021b), it is worth noting that amino-related metabolism was also affected by stress. For instance, the content of γ-aminobutyric acid increased from 1.72 × 10^6^ to 5.8 × 10^6^ under cold stress and reached 6.09 × 10^6^ after the stress disappeared. The gene expression of the γ-aminobutyric synthetic rate-limiting enzyme GADs (CL2552.Contig1_DB,CL2552. Contig5_DB, CL2552.Contig6_DB, and CL2552.Contig7_DB) was significantly increased by cold. Moreover, we also found that low-temperature stress activated the metabolism of polyamine and phenolamide (**Figure 9A**). The key enzymes catalyzing the synthesis of putrescine were largely induced by cold, like *ADC1s* encoding arginine decarboxylase (ID: CL4914.Contig2_DB, CL4914.Contig3_DB) and *PAO2s* encoding polyamine oxidase (CL7418.Contig1_DB, CL12207.Contig1_DB). As a result, the content of putrescine accumulated massively. In a previous study, we found that some phenylpropanoids responded strongly to cold stress. Together with polyamines, they can be catalyzed into phenolamides by *N*-hydroxycinnamoyltransferase. It is worth noting that several phenolamides changed significantly in content under cold. Only *N*′,*N*′′,*N*′′′-*p*-coumaroyl-cinnamoyl-caffeoyl spermidine accumulated rapidly under stress, while *N*′-*p*-coumaroyl agmatine, *N*′-*p*-coumaroyl putrescine, and *N*′-feruloyl putrescine decreased sharply (**Figure 9A**). Interestingly, all of them gradually recovered to normal levels after the stress disappeared. The changes in these amino compounds also suggested their potential functions and importance in stress tolerance.

According to the conserved domain HXXXD, some putative *N*-hydroxycinnamoyltransferases were identified (**Figure 9A**). Combined with the annotation of the database, these enzymes are divided into spermidine hydroxycinnamoyltransferase, omega-hydroxypalmitate *O*-feruloyltransferase, hydroxycinnamoyl-CoA: shikimate/quinate hydroxycinnamoyltransferase, and agmatine coumaroyltransferase. Their encoding genes responded differently to cold, suggesting that the expressions of these genes affected the contents of phenolamine.

**Figure 10:**
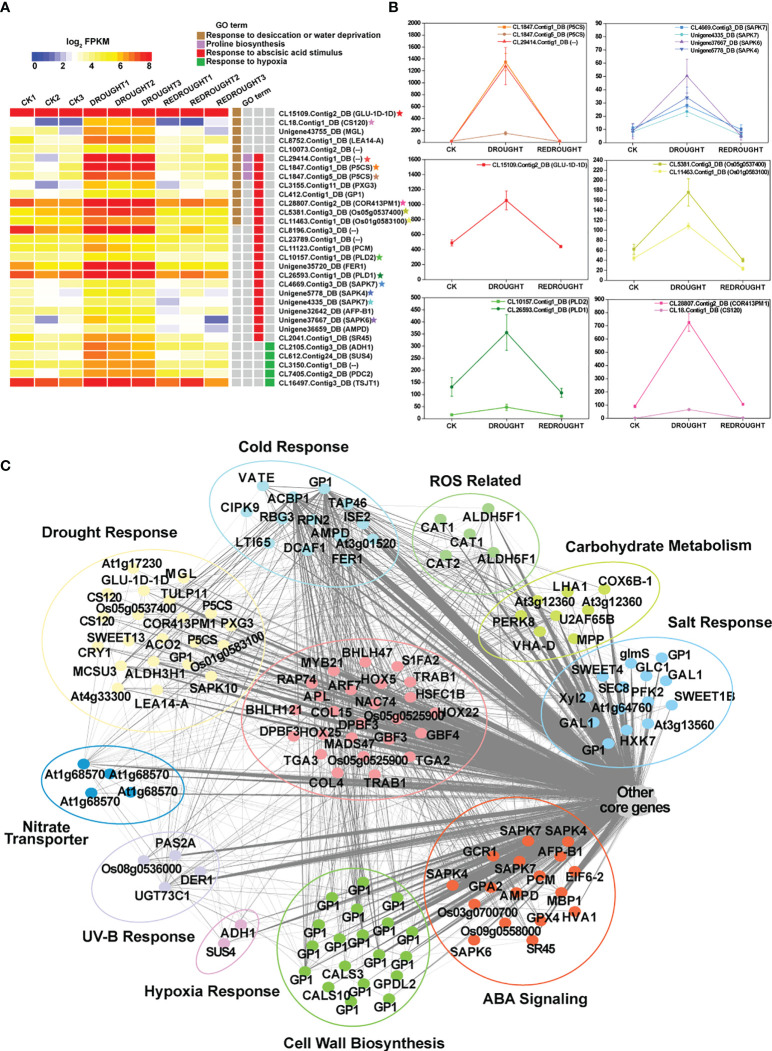
The function, expression pattern, and network of DMEblue hub genes under drought. **(A)** Expression profiles of the representative DMEblue genes (**Supplementary Figure S4**). Some genes marked with stars as the characterization of GO term show expression trends in line graphs. **(B)** The line graphs of some function genes with a star mark related to the drought stress response. The horizontal axis denotes the sampling points, and the vertical coordinates reflect the gene expression level (FPKM value). **(C)** Different functions of DMEblue hub genes.

In drought-related datasets (CK, Drought, and ReDrought), 14,346 unigenes were clustered into 32 drought-related modules (DMEs) (**Supplementary Figure S6**; **Supplementary Table S5-2**). According to the annotation, most genes responsive to drought and ABA were present in DME-blue (**Supplementary Figure S6**), such as *ADH1*, *LEA14-A*, *P5CS*, and *SAPK4* (**Figures 10A, B** **)**. The 853 hub genes were the core of 4,086 genes in DME-blue and were involved in cell wall biosynthesis, nitrogen transport, hypoxia, UV-B response, and other biological processes (**Figure 10C**). In the core genes, a total of 25 transcription factors were identified, and most of them were from the bZIP family (GBF3, GBF4, TGA2, TGA3, TRAB1, and DPBF3). HSFC1B (CL6331.Contig3_DB) was induced to express and was silenced after the stress disappeared.

These 32 DMEs showed different relationships with the 203 metabolites whose contents changed under drought (**Supplementary Figure S8**; **Supplementary Table S6-2**). A total of 195 metabolites remained highly correlated with DME-blue. Among the 59 metabolites positively correlated (**Supplementary Figure S8**; **Supplementary Table S6-2**) were 19 flavonoids (luteolin *O*-hexosyl-*O*-pentoside, luteolin 6-C-hexoside, 8-C-hexosyl-*O*-hexoside, *O*-methylnaringenin-C-pentoside, etc.), nine carbohydrates (D-glucose 6-phosphate, glucose-1-phosphate, sucrose, etc.), six amino acids and derivatives (proline, L-homocitrulline, D-erythro-sphinganine, etc.), four organic acids (L-malic acid, *p*-coumaroyl quinic acid *O*-glucuronic acid, anisic acid *O*-feruloyl hexoside, etc.), and others (coixol, phytocassane C, dulcitol, etc.). In the 136 metabolites with a high negative correlation, there were 66 flavonoids (isosakuranetin, baicalin, prunetin, acacetin, etc.), 16 lipids (LysoPC 20:4, LysoPC 18:0, LysoPC 18:1, LysoPE 18:2, etc.), 12 organic acids (3-*O*-*p*-coumaroyl shikimic acid, 5-*O*-*p*-coumaroyl shikimic acid, 4-hydroxybenzaldehyde, etc.), 11 phenylpropanoids (3-hydroxy-4-methoxycinnamic acid, ferulic acid, caffeate, *p*-coumaric acid, etc.), and others (lumichrome, L-ascorbate, L-dencichin, *N*-lauryldiethanolamine, 1-methylguanidine, etc.).

Similarly, amino compound metabolisms, especially amino acid and polyamine metabolisms, were affected by drought stress. *P5CSs* and *PROC*, the enzymes that catalyze proline synthesis, were upregulated, and the abundance of some *P5CSs* surged up to 116 times (**Figure 9B**). As a result, the L-proline content increased from 7.85 × 10^6^ to 57.5 × 10^6^. The metabolic enzymes OAT, ARGAH1, ADC1, etc., linked proline to other amino acids, and polyamine metabolism was slightly regulated by the stress (**Figure 9B**). In the metabolic pathways, arginine, agmatine, and putrescine increased, and only ornithine decreased. Therein, the content of putrescine increased dramatically from 1.3 × 10^6^ to 3.24 × 10^6^. As an important part of amino compounds, the content of phenolamides also fluctuated under drought stress. Among the three phenolamides with significant changes, *N*-hexosyl-*p*-coumaroyl putrescin and *N*-feruloyl spermidine accumulated rapidly, while *N*-*p*-coumaroyl agmatine was dramatically consumed (**Figure 9B**).

## Discussion

### Potential environmental adaptation strategies

Previously, we found that compounds such as carbohydrates, flavonoids, and phenylpropane and genes involved in their synthesis and regulation, like *CCR*s, *MYB*s, *LEA*s, etc., were induced by low temperature and drought in *P. crymophila*. In addition, the accumulation of schisandrin and flavonoids in the phenylpropanoid pathway probably acted as potential stress-resistant substances to improve the tolerance of plants to stresses (Wang et al., 2021b). In this study, by analyzing gene expression patterns and comprehensively correlating metabolomic and transcriptomic results, more details of *P. crymophila’s* responses to cold and drought were revealed, leading to a deeper understanding of its adaptation strategies to alpine environments.

**Figure 11:**
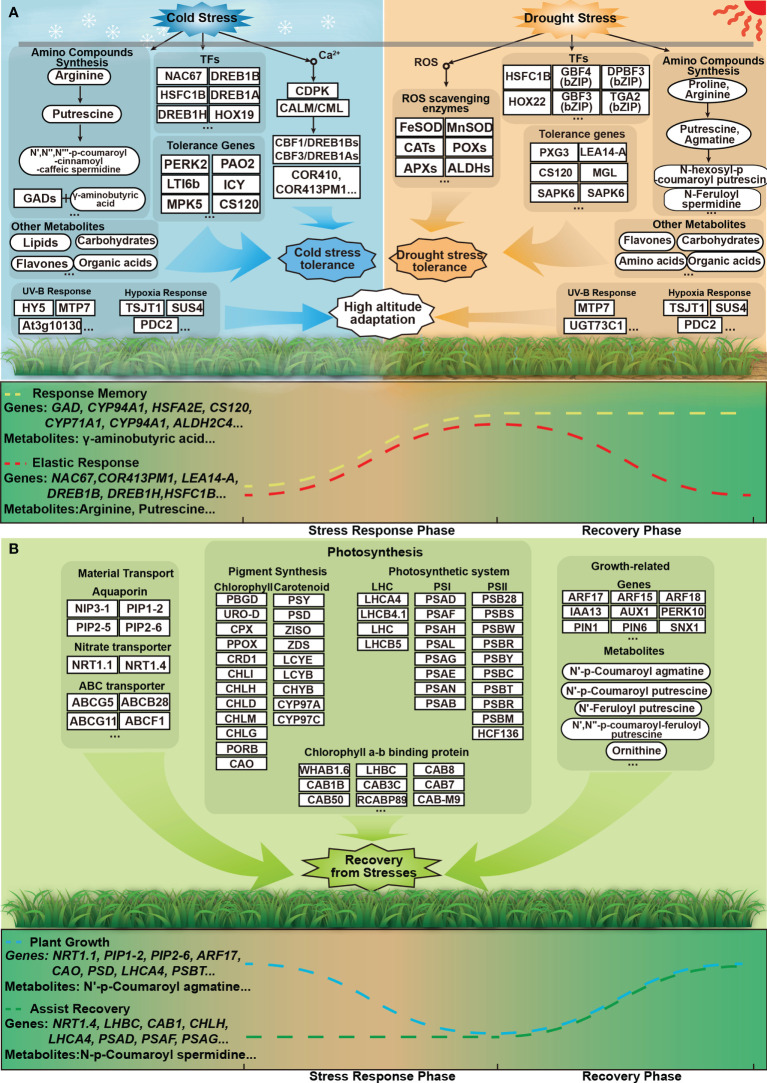
Flexible answer and quick recovery models of *P. crymophila* under cold and drought stresses. **(A)** The stress-adaptive strategies of the induced genes and metabolites. In the stress stage, a large number of response genes are rapidly expressed. Most of these genes and metabolites can be recovered from stress, and some of them are difficult to recover. **(B)** The stress-adaptive strategies of the repressed and unchanged genes and metabolites under adversity. These genes and metabolites involved in material transport, photosynthesis, and plant growth can rapidly restore or improve after stress disappears.

Stresses induced the expression of stress-tolerant genes, inhibited growth-related genes, and most of them quickly returned to normal levels after the stress disappeared. Many crucial genes in a positive response to low temperature and drought stress showed flexible resilience, such as *ABFs*, *CORs*, *CBF/DREBs*, *ALDHs*, *LEA14-A*, etc. Those stress-repressed but quickly recovered genes were mostly related to water, glycerol, carbohydrate, and nitrogen transmembrane transport and other processes. Regardless of the positive or negative response of genes, the rapid recovery of the expressions after the stress disappears is the strong resilience of *P. crymophila* to stresses (**Figure 11**).

In addition, some gene expressions were altered significantly only after the stress was removed. For example, the genes involved in the negative regulation of growth and superoxide anion generation were downregulated after the stress disappeared, while a large number of genes related to photosynthetic pigment synthesis and photosystem composition were rapidly activated. These changes can help plants quickly return to normal growth. These responsive traits also reflected the robust tolerance of *P. crymophila* to cold and drought stresses.

As downstream of regulatory genes, metabolic genes and related metabolites also contribute to plant tolerance. Based on the transcriptome-associated metabolome analysis, we found genes involved in nitrate transport and amino compound metabolism and corresponding amino compounds such as amino acids, polyamines, and phenylamines may also play important roles in supporting robust tolerance in *P. crymophila*.

### Ca^2+^ signaling, ROS scavenging system, and core transcription factor responses

Ca^2+^ and reactive oxygen species (ROS) can act as chemical and electrical signals involved in signal transductions responding to various stresses (Wang et al., 2021a). In *P. crymophila*, we found many genes encoded calcium-related proteins that respond to cold stress, such as Ca^2+^ channel protein ACA, calcium-binding protein CMLs, and kinase CPKs. Only a few genes encoded ROS-scavenging enzymes that responded to drought stress, such as iron superoxide dismutases (FeSODs), manganese superoxide dismutases (MnSODs), catalases (CATs), and ascorbate peroxidases (APXs).

Ca^2+^ is a key signal in the cold response network of plant cells (Guo et al., 2018). The tetraploid cold-tolerant strawberry has a higher expression level of the calcium channel protein so as to ensure the normal transmission of cold stress signals with the fluctuation of calcium ion concentration (Liu et al., 2021a). Calcium-binding protein senses the temperature variation through the change of intracellular calcium level, decodes it as a stress signal, and transmits it downstream as a master switch to control various stress genes (Mahajan and Tuteja, 2005). Therefore, *P. crymophila* may rely on calcium-related proteins to utilize calcium ions as cold stress signals to regulate the expression of downstream stress-responsive genes such as *CBFs* and *CORs* and finally produce physiological and biochemical responses of cold stress defense such as cell wall reinforcement, hypersensitive response, and stomatal closure (Liu et al., 2021a).

ROS can act as a drought stress signal to affect the expression of the downstream genes. However, it also could produce severe negative effects, such as protein denaturation, nucleic acid mutations, and cell damage (Chen and Yang, 2020). Therefore, the ability to scavenge ROS and hydroxyl radicals is an important part of drought tolerance. The SODs are the first line of defense to scavenge O_2_^−^ (Mittler et al., 2004). FeSOD and MnSOD are located separately in chloroplasts and mitochondria, and both can catalyze cytotoxic superoxide anion radicals to generate H_2_O_2_ (Mylona et al., 2007). Furthermore, the H_2_O_2_ is detoxified by the H_2_O_2_ scavenging enzyme group, including CATs, POXs, and APXs, thereby efficiently completing the removal of ROS. These enzymes of the ROS scavenging system protect cells from damage and ensure normal life under stress, which is also the strategy of *P. crymophila* drought adaptation.

The stress signals induced active expression of massive transcription factors, such as *DREBs* under low-temperature stress and *bZIPs* under drought stress. Transcription factors *RAP2-10*, *NAC67*, *HSFC1B*, *HOX22*, *DREB1B*, and *COL4* exhibited similar responses under both stresses, suggesting that their functions under low-temperature and drought stresses are overlapping. Small changes in transcription factors can cause large differences in downstream gene expression. However, *NAC67*, one of the NAC family members, soared rapidly when subjected to both stress and recovered rapidly after the stress disappeared. In rice, the expression of *Os07g12340* (*ONAC067*) was increased with low temperature (Fang et al., 2008; Takasaki et al., 2010). In addition, *EcNAC67* also responded to drought in finger millet (*Eleusine coracana* L.) (Rahman et al., 2016). These transcription factors, especially those located at the core of co-expression networks, may be an important source of plant tolerance.

### Rapid recovery of the photosynthetic system and plateau-specific cross-adaptive genes

Through consensus analysis, we found that the rapid recovery of the photosynthetic system may be the source of resilience. The expression of genes related to the photosynthetic system, such as photosynthetic pigment synthesis, photosystem proteins, etc., increased rapidly after the stress disappeared. The photosynthetic system is an important source of materials and energy and can also be used as a key indicator to measure the impact of stresses. Stress could inhibit the photosynthetic rate and impair the absorption, transfer, and transformation of light energy in leaves, resulting in photoinhibition (Liu et al., 2019). Reportedly, the photosynthetic gene expressions and photosynthesis in maize were inhibited by drought and low-temperature stresses but can be recovered after drought stress disappeared, while it was difficult to recover and even withered after low-temperature stress (Guo et al., 2021). The sensitivity of the photosynthetic system results in low cold tolerance in maize.

Although cold and drought similarly repressed the expression of photosynthetic genes in *P. crymophila*, unlike other plants, these repressed genes can be rapidly recovered after the stress disappeared, even under freezing stress. Furthermore, during the recovery phase, more photosynthetic system genes were activated, and the plants remained green and vigorous in both severe cold and arid environments. In contrast to cold stress assaying, the cool-season forage perennial ryegrass cv. Mathilde, which has cold tolerance, can endure −5°C but was frozen to death when exposed to −12°C. However, *P. crymophila* can stand the chilling stress. All these characteristics of *P. crymophila* reflect its superior resilience.

Furthermore, we also found that both cold and drought activated the expression of genes that responded to UV-B and hypoxia. The expression of *SUS4*, *ADH1*, and *PDC2* was induced by hypoxia. In *sus4* mutants, root growth was retarded under hypoxia (Liu et al., 2017). ADH1 and PDC2 also play important roles in hypoxia stress response as important tolerant genes (Yang, 2014; Gravot et al., 2016). UVR8, RUP1, HY5, and UGT73C1 were identified as key players in UV-B response (Jiang et al., 2012; Lee, 2016) in *P. crymophila* under cold and drought stress. After experiencing a certain kind of adversity, the tolerance of plants to other stresses will also be improved, and this adaptation between different adversities is called cross-adaptation. Due to the special growth habitat of *P. crymophila*, it may have produced special cross-resistance consistent with the high-altitude environment. Similar features were found in the high-altitude perennial *Arabis alpine*, which is more tolerant to UV-B-induced oxidative stress than its relative *Arabidopsis thaliana* (Ozgur et al., 2021). Therefore, this particular cross-adaptation is the result of long-term growth at high altitudes. Through the initial perception of abiotic stresses such as low temperature and drought, genes related to hypoxia and UV-B were stimulated, thereby allowing *P. crymophila* to adapt to complex and harsh high-altitude conditions.

### Nitrate transport and amino compound metabolism

Genes involved in nitrate transport and amino compound metabolism were also affected by cold and drought stresses. The transport of nitrate and nitrogen compounds is mainly through NRT1/PTR family proteins (Wang et al., 2012). The stresses mainly affect the expression of NRT1.1, NRT1.3, and NRT1.4 genes. After the stresses disappeared, the expressions of NRT1.1s (stress-inhibited expression) and NRT1.3s (stress-promoted expression) rapidly recovered, and NRT1.4s increased rapidly. The root-specific NRT1.1s is a dual-affinity transporter involved in soil nitrate uptake and auxin transport (Fang et al., 2021), which suggested that NRT1.1 is associated with the growth of plants. NRT1.3s are highly expressed in flowers, leaves, and stems. These genes are involved in nitrogen transport, polyamine metabolism, and chlorophyll synthesis (Tong et al., 2016). The petiole-specific NRT1.4s was shown to be a low-affinity nitrate transporter. The different responses of the three transporter genes may be related to the metabolic changes of amino compounds under stress, which confer *P. crymophila* with strong adaptability to low temperatures and drought.

Amino acids and polyamines are common and important amino compounds in plants with a variety of important biological functions, including abiotic stress responses. Phenolamines are also increasingly important as functional amino compounds for plants to adapt to adversity, and their functions are closely related to polyamines and amino acids (Macoy et al., 2015). It is of great significance to study the functions of amino acids, polyamines, and phenolamines in plant growth and development and stress.

Changes in the expression of metabolism-related genes affect the anabolism of small molecules. Cold-induced expression of arginine decarboxylase gene (*ADC1*) promoted the synthesis of putrescine, doubling its content in *P. crymophila*. Reportedly, after exposure to cold stress, ADC1 was uniquely enhanced, and exogenous application of putrescine or overexpression of the ADC1 gene could improve potato cold tolerance (Kou et al., 2018). Furthermore, as putrescine accumulated, arginine levels not only did not drop but nearly tripled. Exogenous application of arginine reduced peroxidase activity, malondialdehyde content, and electrolyte leakage and enabled tomato to relieve plant frostbite, but treatment with arginase inhibitors aggravated the damage (Zhang et al., 2010). Thus, arginine accumulation can impart a higher cold tolerance to plants. Furthermore, coumaroyl, feruloyl, caffeoyl, etc. can be added to polyamines to form different phenolamines by BAHD acyltransferases. Under freezing stress, only the content of *N*′,*N*′′,*N*′′′-*p*-coumaroyl-cinnamoyl-caffeic spermidine significantly increased fivefold, which suggested that this particular phenolamine may be closely related to tolerance to abiotic stress. Currently only found in *Salix viminalis*, this special spermidine contributes to the nonspecific protection of plants after phenanthrene treatment (Xia et al., 2021). In addition, some phenolamines like *N*′-*p*-coumaroyl agmatine, *N*′-*p*-coumaroyl putrescine, and *N*′-feruloyl putrescine decreased after low-temperature stress. Some of them were detected during the germination and growth of rice seeds, which are related to growth (Park et al., 2009). As the stress subsided, the abovementioned gene expression levels and small molecule content quickly returned to normal. Therefore, the rapid response and recovery of polyamine and phenolamide metabolism are also important sources of cold tolerance in *P. crymophila*. On another front, after the low temperature disappeared, γ-aminobutyric acid (GABA) and its synthase gene GADs remained at high levels, which may be related to the formation of cold domestication in plants. GABA plays an important role in the cold domestication of mulberry, spinach (*Spinacia oleracea*) (Yoon et al., 2017), barley, and wheat (Mazzucotelli et al., 2006).

The effects of drought stress on amino compound metabolism can be traced back to proline. The abundance of *P5CS*, encoding a key enzyme that catalyzes the synthesis of proline, increased by more than 100 times under drought, and the content of proline also increased by nearly eight times. Proline as an efficient osmotic regulator can improve plant tolerance to drought and play an important role in stress (Per et al., 2017). Ornithine and arginine cannot only participate in plant polyamine metabolism but also improve plant tolerance to drought and salt stress (Anwar et al., 2018; Hussein et al., 2019). When exposed to drought, the content of arginine increased in *P. crymophila*, while ornithine decreased. It is speculated that part of ornithine was used for the synthesis of proline (Díaz et al., 2005). Regarding polyamines, the contents of agmatine and putrescine accumulated. Both of them play an important role in improving plant drought tolerance (Juzoń et al., 2017). In addition, the changes and functions of phenolamine are also worthy of attention. Different from low-temperature stress, drought accumulated the content of *N*-hexosyl-*p*-coumaroyl putrescin and *N*-feruloyl spermidine, and their functions in plants have not yet been reported. These special metabolites are speculated to be drought-tolerant substances in plants, which deserve further verification in the future.

Although there were massive differences in amino compound metabolism under cold and drought stresses, some commonalities could also be found. Arginine and putrescine were abundantly accumulated, while *N*-*p*-coumaroyl agmatine was consumed, which indicated their functions were crossed under low-temperature and drought stress. Taken together, these results suggested that the metabolism of amino acids, polyamines, and phenolamides contributes to plant adaptation to stressful environments.

Interestingly, some neural-related metabolites were detected, such as serotonin, melatonin, and acetylcholine (ACh). Although these compounds did not change significantly under stress, it is speculated that their responses probably be as fast and transient as in animals, resulting in no significant changes being detected. Plant neurobiology is an emerging field of research, which shows the roles of nerve signal substances in various plant processes, including environmental stress adaptation (Michmizos and Hilioti, 2019).

## Conclusion

*Poa crymophila* cv. Qinghai is a forage species grown on the Qinghai–Tibet Plateau, and its special environment has given it a strong and special adaptation to quickly cope with cold and drought, as well as the ability to quickly recover its growth after the stresses have been removed. We previously mainly identified genes involved in the phenylpropanoid pathway, as well as secondary metabolites such as schisandrin, small sugars, and flavone. In this study, further in-depth analysis and clustering of gene expression patterns revealed that pathways such as Ca^2+^ signaling (*ACA*, *CML*, *CPK*, etc.), ROS scavenging system (*SOD*, *CAT*, *APX*, etc.), core transcription factors (*DREB1H*, *NAC67*, *HSFC1B*, etc.), nitrate transport and amino compound metabolism (*NRT1.3*, *PAO2*, *ADC*, etc.), as well as photosynthesis-related genes, respond fast and flexibly to stress. Meanwhile, amino acids (arginine, asparagine, proline, L-homocitrulline, etc.), lipids (LysoPC 16:1, 9-HOTrE, LysoPC 20:4, LysoPC 18:0, etc.), organic acids (azelaic acid, γ-aminobutyric acid, L-Malic acid, etc.), and other compounds (nandrolone, azadiradione, coixol, phytocassane C, dulcitol, etc.) actively respond to stresses. The flexible response of a large number of genes and metabolites under stress, and the rapid recovery after stress relief, endows *P. crymophila* with robust cold and drought tolerance.

## Data availability statement

The original contributions presented in the study are included in the article/**Supplementary Material**. Further inquiries can be directed to the corresponding author.

## Author contributions

X-RM and X-YL conceived this study, designed the experimental plan, analyzed data, and drafted and revised the manuscript. YW designed the experimental plan and participated in data analyses. X-YH, YC, and C-XL participated in sample preparation, treating, collecting, and total RNA extracts. All authors contributed to the article and approved the submitted version.

## Funding

This work was supported by the Key Research and Development Program of Sichuan, China (Grant No.2019YFN0017) and the National Natural Science Foundation of China (Grant No.31502003).

## Conflict of interest

The authors declare that the research was conducted in the absence of any commercial or financial relationships that could be construed as a potential conflict of interest.

## Publisher’s note

All claims expressed in this article are solely those of the authors and do not necessarily represent those of their affiliated organizations, or those of the publisher, the editors and the reviewers. Any product that may be evaluated in this article, or claim that may be made by its manufacturer, is not guaranteed or endorsed by the publisher.

## Supplementary material

Supplementary Figure 1 Analysis of network topology for various soft-thresholding powers. **(A)** cold related co-expression network; **(B)** drought related co-expression network.

Supplementary Figure 2 Pearson’s correlation coefficient between samples.

Supplementary Figure 3 The 2D and 3D PCA results of all unigenes and filtered unigenes. **(A, B)** The 2D PCA results of all unigenes and filtered unigenes, respectively; **(C, D)** The 3D PCA results of all unigenes and filtered unigenes, respectively; **(A, C)** reflects the expression profiles of unigenes before filtered, and **(B, D)** shows the expression of filtered unigenes. In the legend, CK represents control group; **(C)** denotes cold stress group; **(D)** represents drought stress group; RC represents cold recovery group; RD denotes drought recovery group. The axes represents the dimension reduction axis, and the number represents the percentage interpretation rate of the axis.

Supplementary Figure 4 The Venn diagrams of differential expressed genes. **(A)** Cold stress dataset; **(B)** Drought stress dataset.

Supplementary Figure 5 Cold related weighted gene co-expression network. **(A)** Clustering dendrogram of genes, with dissimilarity based on topological overlap, together with assigned module colors; **(B)** Visualization of the eigengene network representing the relationships among the modules and the content of D-glucose-6phosphate; **(C)** Visualizing the gene network using a heatmap plot.

Supplementary Figure 6 Drought related weighted gene co-expression network. **(A)** Clustering dendrogram of genes; **(B)** Visualization of the eigengene network; **(C)** Visualizing the gene network using a heatmap plot.

Supplementary Figure 7 Correlations between cold-responsive metabolites and different modules. The relationship from negative to positive is represented by the color from blue to red.

Supplementary Figure 8 Correlations between drought-responsive metabolites and different modules. The relationship from negative to positive is represented by the color from blue to red.

Supplementary Table 1 Gene expressions of P. crymophila and expression differences

Supplementary Table 2 Gene Expression Clusters.

Supplementary Table 3 The results of consensus analysis.

Supplementary Table 4 The results of module preservation analysis.

Supplementary Table 5 The results of cold and drought co-expression network.

Supplementary Table 6 The association results between differential metabolomes and co-expression networks.
