# Supplementary figures and images for: Flexible response and rapid recovery strategies of the plateau forage Poa crymophila to cold and drought
Source: Front Plant Sci. 2022 Nov 8;13:970496. doi: 10.3389/fpls.2022.970496 (PMC9681527; doi:10.3389/fpls.2022.970496)

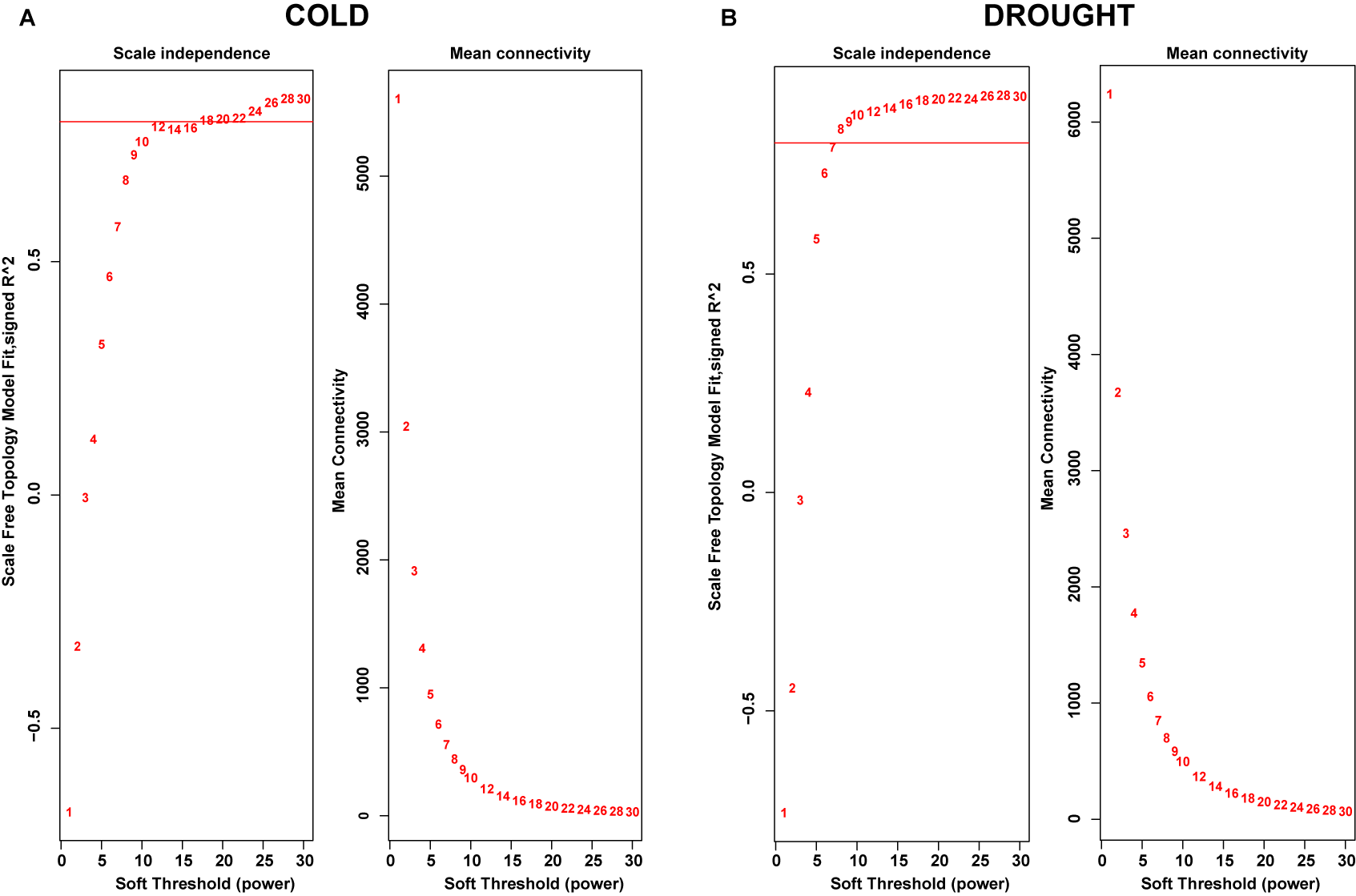

Supplement: Supplementary Figure 1 — Analysis of network topology for various soft-thresholding powers. (A) cold related co-expression network; (B) drought related co-expression network. [file Image_1.tif]

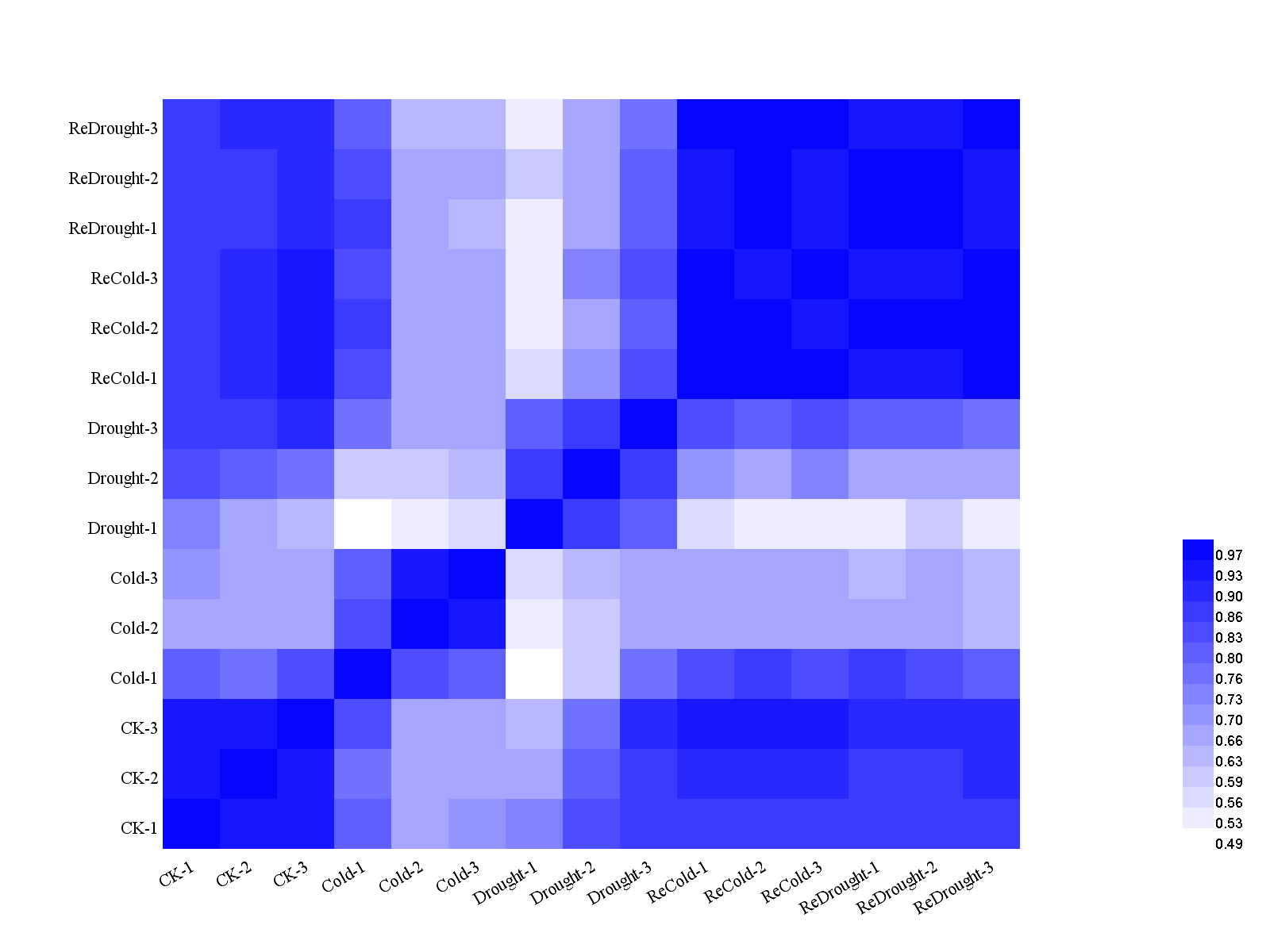

Supplement: Supplementary Figure 2 — Pearson’s correlation coefficient between samples. [file Image_2.tiff]

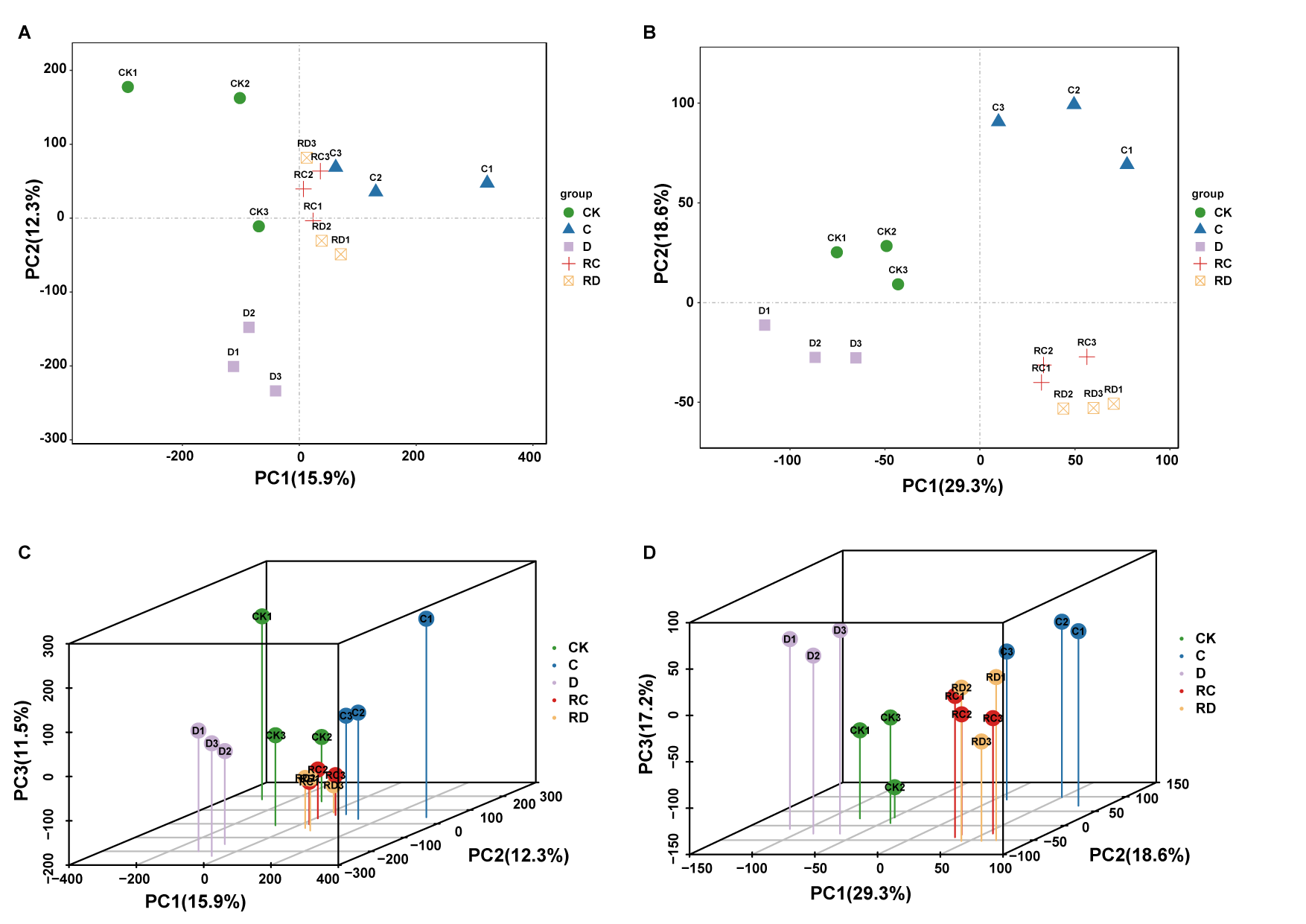

Supplement: Supplementary Figure 3 — The 2D and 3D PCA results of all unigenes and filtered unigenes. (A, B) The 2D PCA results of all unigenes and filtered unigenes, respectively; (C, D) The 3D PCA results of all unigenes and filtered unigenes, respectively; (A, C) reflects the expression profiles of unigenes before filtered, and (B, D) shows the expression of filtered unigenes. In the legend, CK represents control group; (C) denotes cold stress group; (D) represents drought stress group; RC represents cold recovery group; RD denotes drought recovery group. The axes represents the dimension reduction axis, and the number represents the percentage interpretation rate of the axis. [file Image_3.tif]

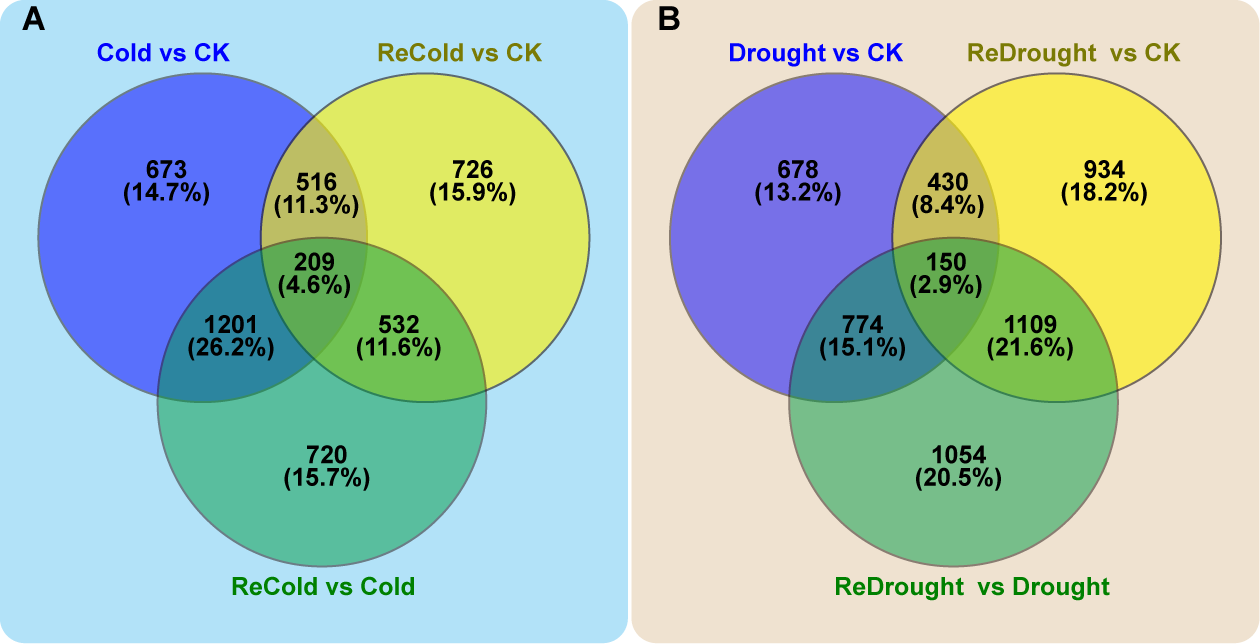

Supplement: Supplementary Figure 4 — The Venn diagrams of differential expressed genes. (A) Cold stress dataset; (B) Drought stress dataset. [file Image_4.tif]

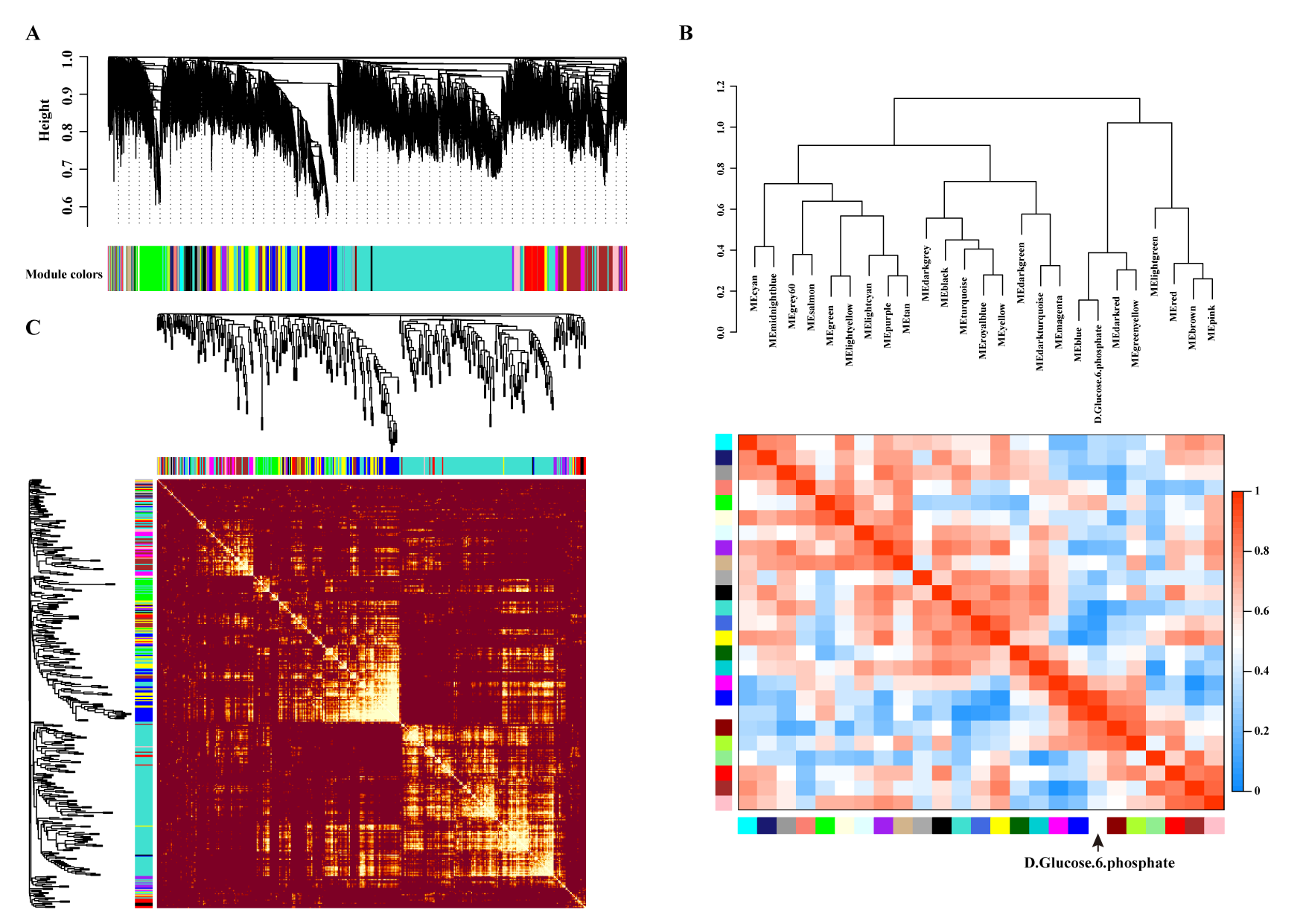

Supplement: Supplementary Figure 5 — Cold related weighted gene co-expression network. (A) Clustering dendrogram of genes, with dissimilarity based on topological overlap, together with assigned module colors; (B) Visualization of the eigengene network representing the relationships among the modules and the content of D-glucose-6phosphate; (C) Visualizing the gene network using a heatmap plot. [file Image_5.tif]

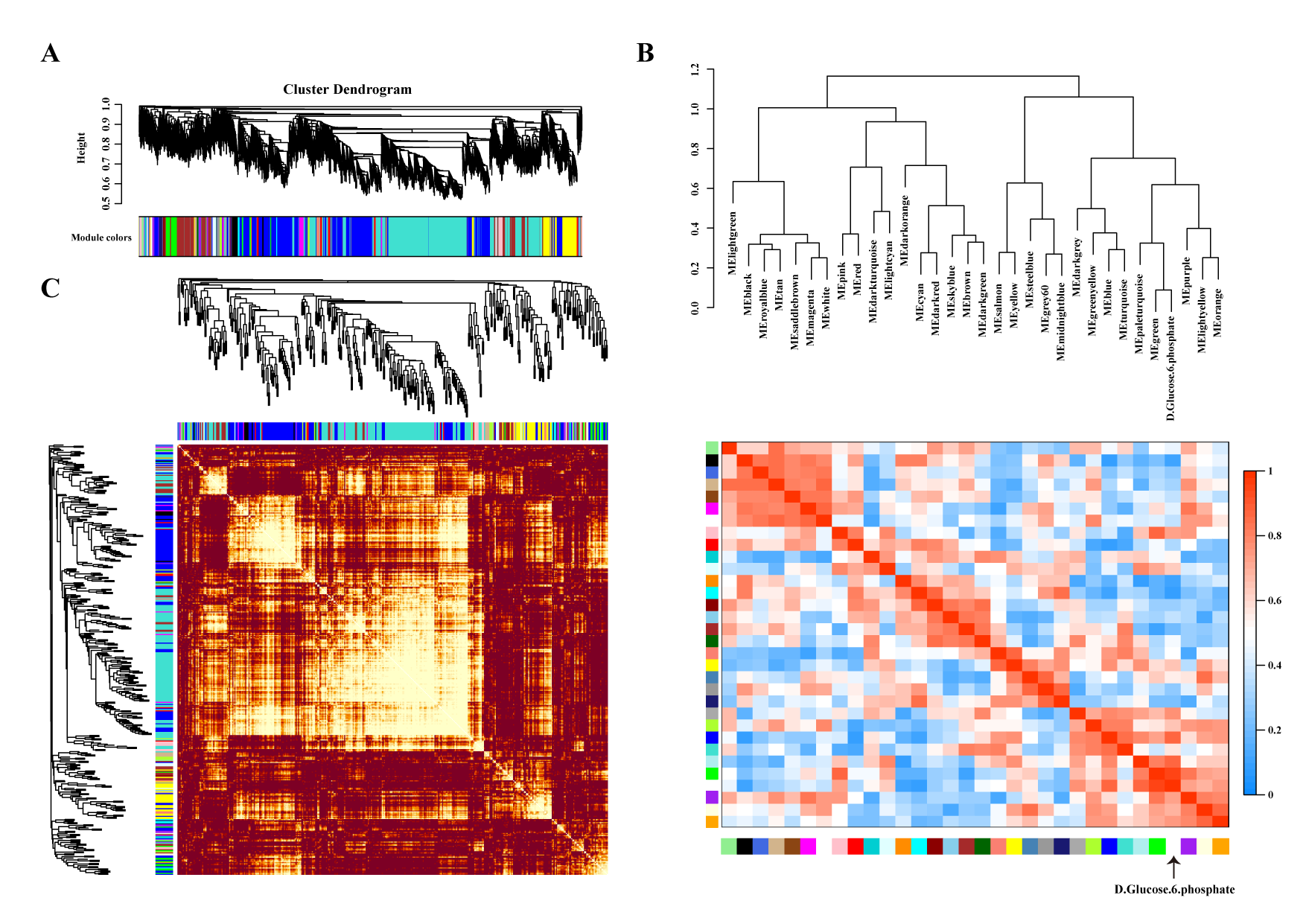

Supplement: Supplementary Figure 6 — Drought related weighted gene co-expression network. (A) Clustering dendrogram of genes; (B) Visualization of the eigengene network; (C) Visualizing the gene network using a heatmap plot. [file Image_6.tif]

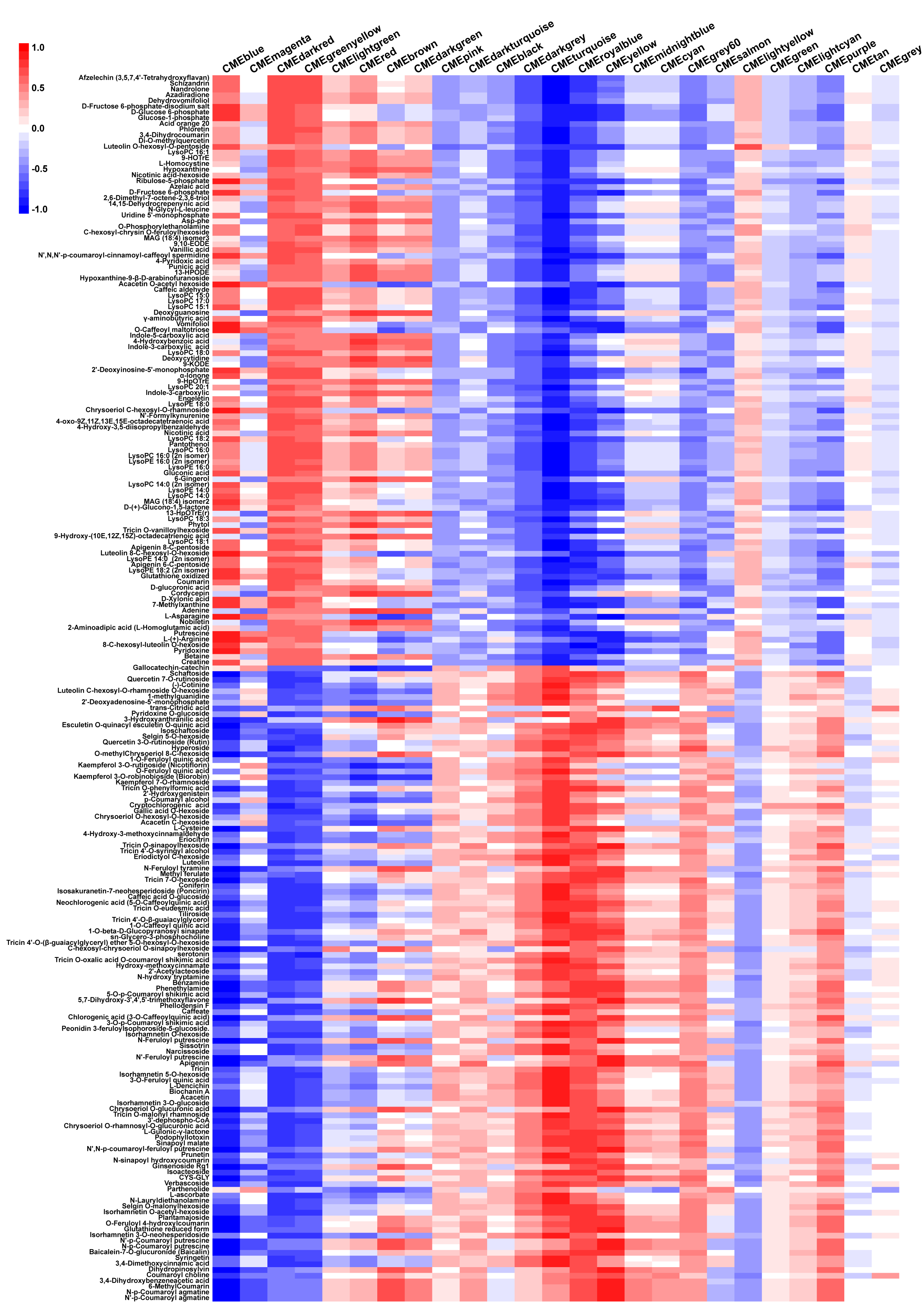

Supplement: Supplementary Figure 7 — Correlations between cold-responsive metabolites and different modules. The relationship from negative to positive is represented by the color from blue to red. [file Image_7.tif]

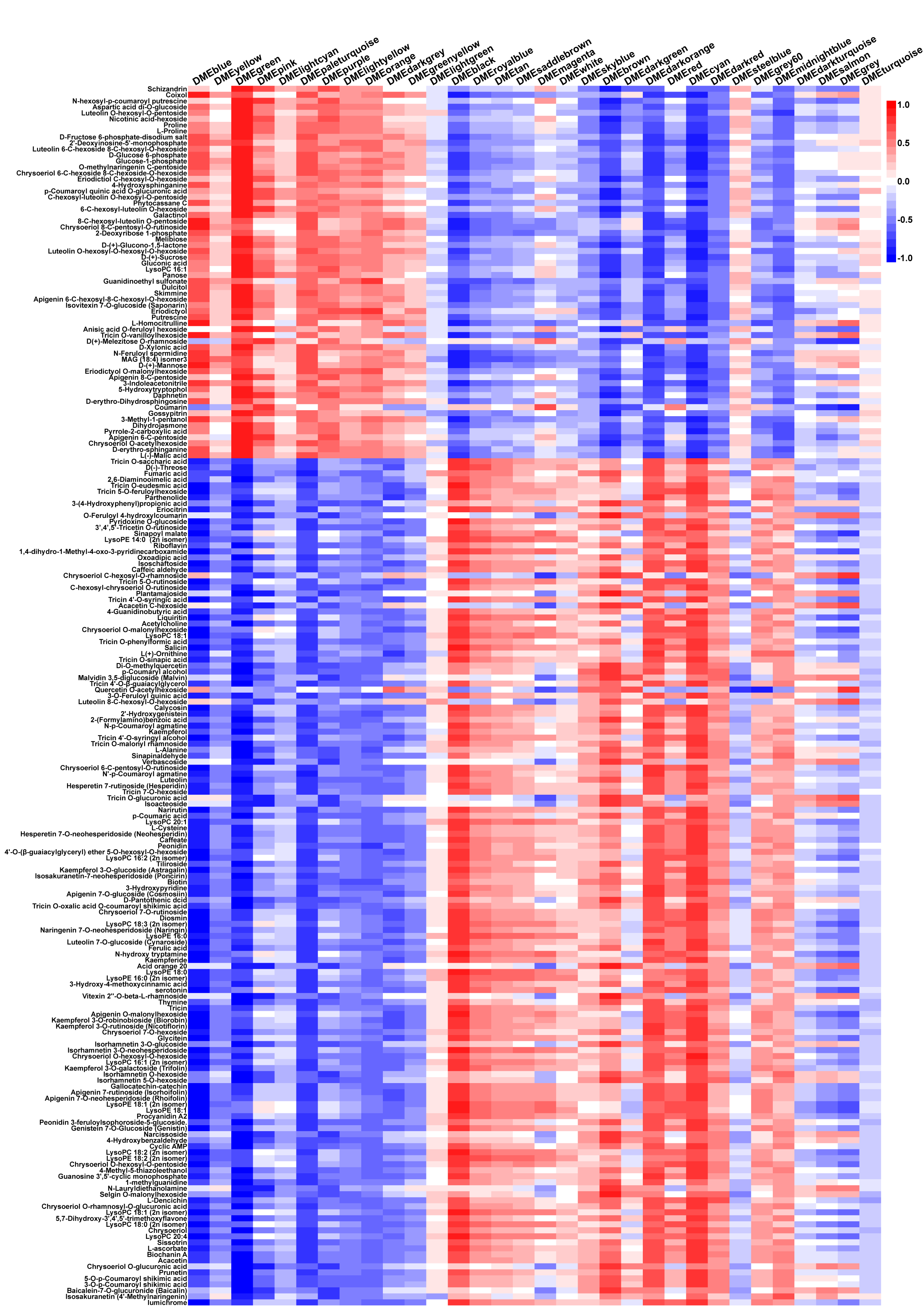

Supplement: Supplementary Figure 8 — Correlations between drought-responsive metabolites and different modules. The relationship from negative to positive is represented by the color from blue to red. [file Image_8.tif]
